# Short-spored *Subulicystidium* (Trechisporales, Basidiomycota): high morphological diversity and only partly clear species boundaries

**DOI:** 10.3897/mycokeys.35.25678

**Published:** 2018-06-27

**Authors:** Alexander Ordynets, David Scherf, Felix Pansegrau, Jonathan Denecke, Ludmila Lysenko, Karl-Henrik Larsson, Ewald Langer

**Affiliations:** 1 Department of Ecology, FB 10 Mathematics and Natural Sciences, University of Kassel, Heinrich-Plett-Strasse 40, 34132 Kassel, Germany; 2 Natural History Museum, University of Oslo, P.O. Box 1172 Blindern, 0318 Oslo, Norway

**Keywords:** basidiospores, biodiversity, biometry, crystals, cystidia, DNA barcode, encrustation, genetic distance, internal transcribed spacer, large subunit, species delimitation, taxonomy

## Abstract

Diversity of corticioid fungi (resupinate Basidiomycota), especially outside the northern temperate climatic zone, remains poorly explored. Furthermore, most of the known species are delimited by morphological concepts only and, not rarely, these concepts are too broad and need to be tested by molecular tools. For many decades, the delimitation of species in the genus *Subulicystidium* (Hydnodontaceae, Trechisporales) was a challenge for mycologists. The presence of numerous transitional forms as to basidiospore size and shape hindered species delimitation and almost no data on molecular diversity have been available. In this study, an extensive set of 144 *Subulicystidium* specimens from Paleo- and Neotropics was examined. Forty-nine sequences of ITS nuclear ribosomal DNA region and 51 sequences of 28S nuclear ribosomal DNA region from fruit bodies of *Subulicystidium* were obtained and analysed within the barcoding gap framework and with phylogenetic Bayesian and Maximum likelihood approaches. Eleven new species of *Subulicystidium* are described based on morphology and molecular analyses: *Subulicystidium boidinii*, *S. fusisporum*, *S. grandisporum*, *S. harpagum*, *S. inornatum*, *S. oberwinkleri*, *S. parvisporum*, *S. rarocrystallinum*, *S. robustius*, *S. ryvardenii* and *S. tedersooi*. Morphological and DNA-evidenced borders were revised for the five previously known species: *S. naviculatum, S. nikau*, *S. obtusisporum*, *S. brachysporum* and *S. meridense*. Species-level variation in basidiospore size and shape was estimated based on systematic measurements of 2840 spores from 67 sequenced specimens. An updated identification key to all known species of *Subulicystidium* is provided.

## Introduction

The genus *Subulicystidium* was created by Parmasto (1968) to accommodate corticioid fungi with long subulate or sword-like cystidia with a unique morphology. The smooth thick crystalline sheath of cystidia is covered with two chains of the bow-tie-shaped crystals, which are seen in the light microscope as four chains of rectangular crystals along the cystidium body (Jülich 1975, Keller 1985). Other morphological characters of the genus are resupinate arachnoid fruit-bodies, loosely interwoven hyphae with constant clamps and suburniform basidia (Oberwinkler 1977, Duhem and Michel 2001). Repetobasidia were also noted by some authors (Jülich 1968, Liberta 1980). The genus belongs to the order Trechisporales K.H.Larss., though its relationship with the other genera within the family Hydnodontaceae Jülich remains unclear (Larsson 2007, Telleria et al. 2013). Fruit-bodies of *Subulicystidium* are found on decayed wood or other plant debris at the forest floor but exact nutrition mode of the genus is not known (Hibbett et al. 2014).

Currently, nine species are recognised based on morphological features (Index Fungorum 2018). The generitype *S. longisporum* (Pat.) Parmasto is often reported and mapped in mycodiversity surveys worldwide (e.g. see Subulicystidium 2017). In contrast, other species are still known either from the type locality only (*S. curvisporum* Gorjón, Gresl. & Rajchenb.) or from a few localities: *S. brachysporum* (P.H.B. Talbot & V.C. Green) Jülich, *S. cochleum* Punugu, *S. meridense* Oberw., *S. naviculatum* Oberw., *S. nikau* (G. Cunn.) Jülich and *S. obtusisporum* Duhem & H. Michel (Punugu et al. 1980, Gorjón et al. 2012). Some species records represent more than one continent but in all cases these reports are based on a morphological species concept (Boidin and Gilles 1988, Duhem and Michel 2001, Volobuev 2016).

Species delimitation in *Subulicystidium* has remained challenging. Basidiospore size and shape were traditionally used as the main discriminating characters, while other microscopic structures of fruit-bodies were considered as generally invariable (Oberwinkler 1977, Boidin and Gilles 1988, Duhem and Michel 2001). However, overlap of spore size between species is reported, as well as high morphological variability of the spores within single collections (Liberta 1980, Hjortstam and Ryvarden 1986). This has led to doubts on the identity of some taxa. For example, Liberta (1980) regarded *S. longisporum* as a highly variable “species complex” and that *S. brachysporum* (P.H.B. Talbot & V.C. Green) Jülich and *S. meridense* Oberw. should not be accepted until additional data for species limit evaluation became available (Oberwinkler 1977, Liberta 1980).

Despite the general progress in molecular identification of fungi during the last three decades (Kõljalg et al. 2013), almost no data on the genetic diversity within *Subulicystidium* have been published and the genus remains poorly represented in all kinds of molecular studies. Currently available public sequences are usually identified to genus level only or even just named “Trechisporales”. Public sequences from fungal fruit-bodies annotated to the species level are few (Volobuev 2016).

During recent decades, extensive collections of *Subulicystidium* were made by us in Paleo- and Neotropics. In this paper, 11 new species of *Subulicystidium* are reported based on morphological and molecular evidence (similarities and phylogenies based on rDNA ITS and 28S sequences). The concepts of five previously known species are clarified and the possibility of species presence on several continents is verified. In the current study, we focus on rich material with relatively short basidiospores, i.e. non-acicular and often less than 10 µm long, thus leaving out *S. longisporum*-like material for a future study.

## Materials and methods

### Assembling dataset

In this study, we examined 144 herbarium specimens of the genus *Subulicystidium*, which were collected in several regions of Paleotropics (Réunion Island, Madagascar, Africa, South-East Asia) and Neotropics (Caribbean region, various countries of South America). This material was collected during the last six decades, with the oldest collection (PDD13816) from 1954 and the most recent ones from 2015 (e.g. KAS:L 1860). Collections are preserved in the following herbaria: O (Natural History Museum, Oslo University, Norway), GB (Gothenburg University, Sweden), MG (Museu Paraense Emílio Goeldi, Belém, Brasil), SP (Instituto de Botânica, São Paulo, Brasil), KAS (University of Kassel, Germany), FR (Senckenberg Research Institute and Natural History Museum, Frankfurt am Main, Germany) and LY (University of Lyon, France). We examined also holotype specimens of *Subulicystidium meridense* Oberw. (TUB, Tübingen University, Germany), *S. nikau* (G. Cunn.) Jülich (PDD, New Zealand Fungal Herbarium, Landcare Research, Auckland) and the collection of *S. allantosporum* Boidin and Gilles ad interim (Boidin and Gilles 1988) from LY. Attempts to obtain the type specimen of *S. brachysporum* from PREM (Plant Protection Research Institute, Queenswood, South Africa) were not successful.

For a better biodiversity data availability and reusability, in Suppl. material 1, the table with detailed information on all 144 specimens examined is provided. If missing on the original specimen labels, data on higher-and lower rank administrative units were mined and added to the corresponding columns in Suppl. material 1. The table also includes geographic coordinates for each specimen in decimal degrees format (DD) with minus signs used to indicate southern and western hemispheres. Originally, geographic coordinates were available for 65 specimens. For the other 69 specimens, an attempt to estimate the coordinates from a map was made using resources Google Maps (via http://www.gpskoordinaten.de/), OpenStreetMap (https://www.openstreetmap.org) and the georeferencing calculator of Wieczorek and Wieczorek (2015). In ten specimens, the locality was not precisely indicated to estimate the coordinates. The manner, in which coordinates were obtained, was specified for each specimen in the Suppl. material 1.

Field data and photos of recent collections from Réunion Island (stored in FR and KAS) are accessible via PlutoF workbench (Abarenkov et al. 2010) under the project “Ordynets_Fungi of Reunion Island” and as a part of GBIF occurrence dataset of the Senckenberg herbarium FR (Senckenberg 2018).

### Analysing microscopic traits

Sections from dried herbarium specimens were examined in 3% aqueous solution of potassium hydroxide (KOH) mixed with 1% aqueous solution of Phloxine, using 100× immersion oil lens of a Leica DM500 light microscope. Images were captured with a built-in ICC 50 HD Camera using Leica Application Suite EZ V.3.2.1 software (Leica Microsystems Ltd., Switzerland). Measurements were done with the software “Makroaufmaßprogramm” from Jens Rüdigs (https://ruedig.de/tmp/messprogramm.htm) and analysed with the software “Smaff” version 3.2 (Wilk 2012). At least 30 basidiospores per specimen were measured where possible for the sequenced specimens or otherwise important collections. When referring to the basidiospore measurement results, abbreviation L was used for the spore length, W for the spore width, Q for the length to width ratio and N x/y for the x number of spores measured from y specimens.

The raw spore measurements were undertaken as follows. First, for each collection, automated search for size outliers was performed with the “Smaff” software (Wilk 2012). To account for the outliers in both spore length and width simultaneously, the parameters of (i) length multiplied by width and (ii) volume were calculated for each spore by the programme. These values, (i) followed by (ii), were checked to represent the outliers in the sample on a 95% probability level, using simultaneously the tests of Verma and Quiroz-Ruiz (2006), David et al. (1954) and Grubbs (1950). Upon their detection, outliers were excluded from the sample as recommended by Wilk (2012). This procedure usually resulted in a better fit of the spore measurements to the normal distribution. The spore measurements after excluding outliers are provided in the Suppl. material 2.

These filtered spore measurements were used to calculate the spore size range of the species. The main range was presented as the interval into which 90% of non-outlier measurements fall, while 5% of the smallest and 5% of the largest non-outlier measurements were included in parentheses. For the species with more than one specimen available, the filtered spore measurements were pooled together and the main range (90% of the data) with 5% of the smallest and 5% of the largest values were defined for this pooled sample. Calculations were done in R version 3.3.3 (R Core Team 2017) and script is available from Ordynets and Denecke (2018). Additionally, for species with at least three sequenced specimens, hypothetical intervals were calculated within which 90% of all existing individuals’ specimen mean values lie. This way of representing basidiospore size variability in species was highly recommended by Parmasto and Parmasto (1987) and Raitviir (1972). These 90% tolerance intervals were calculated for the 90% probability level, with the method of Howe (1969) implemented in the “normtol.int” function of the “tolerance” R package version 1.3.0 (Young 2010). R script for these calculations is available in Ordynets (2018b).

At least 10 basidia and cystidia were measured per specimen and their size variation was presented simply as the range between minimum and maximum values for the pooled measurements of all collections belonging to one species. When basally swollen cystidia was a regular feature, both the largest diameter at the place of swelling and the diameter next to the swelling were noted. The protruding bow-tie crystals were included in the measurements of cystidium diameter. The shape of the cystidium apex followed terminology for sterile hymenial elements of Yurchenko and Wu (2016) and included the following options: tapering, acute and acuminate. Cystidial ornamentation was described as seen under the light microscope.

### DNA extraction, amplification and sequencing

Sequences of two nuclear ribosomal DNA regions were considered in our study: internal transcribed spacer (ITS) and ribosomal large subunit-coding DNA (28S). Sequences were obtained from dried herbarium specimens. Total DNA was isolated according to the protocol of Izumitsu et al. (2012). For that, 1-2 mg of fungal fruit body tissue were suspended in 100 µl TE buffer in a 1.5 ml tube. The tubes were microwaved (700 Watt) for 1 min two times, with a 30 seconds pause while keeping the tubes at room temperature. Tubes were cooled at -20°C for 20 min and centrifuged at 10000 rpm for 5 min. The supernatants were 10 or 100 times diluted and in this form used in PCR.

Primer pairs used to amplify the complete ITS region were ITS1F/ITS4, ITS1/ITS4 and ITS1/ALR0 (White et al. 1990, Gardes and Bruns 1993, Collopy et al. 2001). The D1–D2 domains at the 5' end of 28S were amplified with primer pairs NL1/NL4 (O’Donnell 1992) and less frequently with LR0R/LR5 (Hopple and Vilgalys 1999). PCRs of the collections from Réunion Island (FR and KAS herbaria) were performed as explained in Ordynets et al. (2015). The PCR for the remaining material was performed on 53 µl solution containing 5 µl of extracted DNA and 48 µl Master Mix (BIOLINE GmbH, Luckenwalde, Germany). One Master mix portion contained 30.2 µl H_2_O, 10 µl reaction buffer (30 mM MgCl_2_) coloured with red and orange dyes, 2 µl MgCl_2_ (50 mM), 2 µl dNTPs (6 mM), 2 µl Bovine Serum Albumin (20 µg/µl), 0.8 µl of each forward and reverse primers (25 pM) and 0.2 µl Mango-Taq DNA Polymerase (5 units/µl).

Amplifications were performed in 96-well TGradient Thermocycler (Biometra, Göttingen, Germany). PCR with primer pairs ITS1F/ITS4, ITS1/ALRO and NL1/NL4 was set as initial denaturation at 94°C for 3 min followed by 29 cycles of denaturation 94°C for 30 s, annealing 55°C for 45 s and extension 72°C for 60 s; final elongation was done at 72°C for 7 min. PCR with primer pair LR0R/LR5 differed only in having the annealing temperature as 48°C.

PCR products were checked on 1% agarose gel stained with GelRed fluorescence dye (BIOTIUM, Hayward, CA, USA) in the Transilluminator Biometra Ti5 equipped with BioDocAnalyze software (Biometra GmbH, Göttingen, Germany). PCR products were cleaned with QIAquick PCR Purification Kit according to manufacturer’s instructions (QIAGEN GmbH, Hilden, Germany). Sanger sequencing of purified products was performed in the facilities of the Senckenberg Research Institute and Natural History Museum (Frankfurt am Main, Germany) and by the company GATC Biotech AG (Constance, Germany). The primers used for sequencing were identical to those used for amplification.

The oldest specimen we succeeded to sequence, with regard to both ITS and 28S regions, was from the year 1978 (LR 15483 in O:F 918488). Attempts of DNA amplification from the type specimens of *S. meridense*, *S. nikau* and *S. allantosporum* Boidin ad interim (Boidin and Gilles 1988) were not successful, as well as an attempt to sequence the type specimen of the new species *Subulicystidium ryvardenii* Ordynets, Langer & K.H. Larss. sp. nov. We did not succeed in amplifying two protein-coding genes from any of the specimens: a partial segment (511 bp) of the translation elongation factor 1α (TEF1α) with EF-595f and EF-1160r primer pair (Kauserud and Schumacher 2001), as well as the largest subunit of RNA polymerase II gene (RPB1) with RPB1-Af and RPB1-Cr primer pair (Matheny et al. 2002).

### DNA sequence-based analyses

All sequences obtained in this study went through the standard quality assessment steps outlined by Nilsson et al. (2012). Raw sequence data were processed with Geneious version 5.6.7 (http://www.geneious.com, Kearse et al. 2012). For various sequence format conversions and alignment viewing, Mesquite version 3.40 (Maddison and Maddison 2018), AliView version 1.19 (Larsson 2014) and Seaview version 4 (Gouy et al. 2010) were used.

In this study, 49 sequences of ITS rDNA region and 51 sequences of 28S rDNA region of *Subulicystidium* were generated and submitted to GenBank (Benson et al. 2013). They are available as accessions MH041511-MH041559 for ITS and MH041560-MH041610 for 28S region. Additional ten ITS and six 28S sequences of *Subulicystidium*, earlier available in GenBank and UNITE database (Kõljalg et al. 2013), were downloaded and used in our analyses. Finally, we included sequences of *Brevicellicium exile* (H.S. Jacks.) K.H. Larss. & Hjortstam and *B. olivascens* (Bres.) K.H. Larss. & Hjortstam to serve as an outgroup in our sequence-based analyses. All sequences used in the study are listed with brief metadata in Table 1.

**Table 1.** Ribosomal DNA sequences used in this study with information on voucher specimens. Most sequences are newly generated for this study and ITS and 28S region were sequenced separately. For specimens GB:KHL 14229 and 16100 and TU 124388, single accession number in each case refers to a sequence containing both ITS and 28S regions. Sequences retrieved from other studies are marked with an asterisk. Abbreviation “na” means sequence is not available. In the species *S. brachysporum*, “B” means morphological species concept following Boidin and Gilles (1988), while “T” means the species as described by Talbot (1958).

Species	Locality	Voucher specimen	Collector(s)	GenBank/UNITE accession numbers	
				ITS	28S
*Subulicystidium boidinii*	Costa Rica: Puntarenas	GB:KHL 12830	K.-H. Larsson	MH041537	MH041570
*S. boidinii* (holotype)	Reunion: Saint-Benoit	KAS:L 1584a	M. Striegel	MH041527	na
*S. brachysporum* B	Argentina: Misiones	O:F: 506782	L. Ryvarden	MH041518	MH041572
*S. brachysporum* B	Brazil: Paraiba	O:F: KHL 16100	K.-H. Larsson	MH000599*	MH000599*
*S. brachysporum* B	Brazil: Rondonia	O:F:KHL 15352	K.-H. Larsson	MH041553	MH041576
*S. brachysporum* B	Brazil: Sao Paulo	GB:Hjm 16573	K. Hjortstam	MH041545	MH041596
*S. brachysporum* B	Colombia: Magdalena	O:F: 918493	L. Ryvarden	MH041522	MH041605
*S. brachysporum* B	Costa Rica: Alajuela	GB:KHL 11216	K.-H. Larsson	MH041517	MH041580
*S. brachysporum* B	Jamaica: Cornwall	GB:KHL 10763	K.-H. Larsson	MH041546	MH041598
*S. brachysporum* B	Jamaica: Middlesex	GB:KHL 10566	K.-H. Larsson	na	MH041599
*S. brachysporum* B	Madagascar: Anosy	O:F:KHL 14537	K.-H. Larsson	MH041552	MH041573
*S. brachysporum* B	Puerto Rico: Isabela	GB:KHL 9544	K.-H. Larsson	MH041555	MH041560
*S. brachysporum* B	Puerto Rico: Luquillo	GB:KHL 10406	K.-H. Larsson	MH041543	MH041600
*S. brachysporum* B	Puerto Rico: Luquillo	GB:KHL 10411	K.-H. Larsson	MH041549	MH041601
*S. brachysporum* B	Reunion: Saint Pierre	KAS:L 0134	E. Langer	MH041541	MH041593
*S. brachysporum* B	Reunion: Saint-Benoit	KAS:L 1584b	M. Striegel	MH041544	MH041610
*S. brachysporum* B	Reunion: Saint-Pierre	KAS:L 1147	J. Riebesehl; M. Schroth	MH041542	MH041594
*S. brachysporum* B	Reunion: Saint-Pierre	KAS:L 1498	M. Striegel	MH041526	na
*S. brachysporum* B	Reunion: Saint-Pierre	KAS:L 1795	M. Striegel	MH041525	MH041579
*S. brachysporum* B	Reunion: Saint-Pierre	LY 12293	G. Gilles	MH041550	MH041571
*S. brachysporum* B	Reunion: Saint-Pierre	LY 12772	G. Gilles	na	MH041595
*S. brachysporum* T	Brazil: Rondonia	O:F:KHL 15318	K.-H. Larsson	MH041557	MH041577
*S. brachysporum* T	Brazil: Rondonia	O:F:KHL 15327	K.-H. Larsson	MH041539	MH041603
*S. brachysporum* T	Brazil: Sao Paulo	O:F:LR 24170	D. Pegler; K. Hjortstam; L. Ryvarden	MH041556	na
*S. brachysporum* T	Reunion: Saint-Paul	LY 11378	J. Boidin	na	MH041574
*S. fusisporum*	Costa Rica: Puntarenas	GB:KHL 12761	K.-H. Larsson	MH041536	MH041568
*S. fusisporum*	Puerto Rico: Rio Grande	GB:KHL 9093	K.-H. Larsson	MH041534	na
*S. fusisporum* (holotype)	Puerto Rico: Rio Grande	GB:KHL 10360	K.-H. Larsson	MH041535	MH041567
*S. grandisporum* (holotype)	Costa Rica: Cartago	O:F: 506781	L. Ryvarden	MH041547	MH041592
*S. harpagum*	Colombia: Magdalena	O:F:LR 15736	L. Ryvarden	MH041531	MH041586
*S. harpagum*	Jamaica: Cornwall	GB:KHL 10733	K.-H. Larsson	MH041520	MH041563
*S. harpagum*	Reunion: Saint-Benoit	KAS:L 0244	E. Langer	MH041533	MH041609
*S. harpagum* (holotype)	Reunion: Saint-Pierre	KAS:L 1726a	M. Striegel	MH041532	MH041588
*S. inornatum* (holotype)	Puerto Rico: Rio Grande	GB:KHL 10444	K.-H. Larsson	MH041558	MH041569
*S. longisporum*	Italy: Sicily	TU 124391	A. Saitta	UDB028356*	UDB028356*
*S. longisporum*	Russia: Orel	LE 292121	S. Volobuev	KP268491*	na
*S. longisporum*	Sweden: Skåne	GB:KHL 14229	K.-H. Larsson	MH000601*	MH000601*
*S. meridense*	Brazil: Rondonia	O:F:KHL 15322	K.-H. Larsson	MH041540	MH041602
*S. meridense*	Brazil: Sao Paulo	GB:Hjm 16400	D. Pegler; K. Hjortstam; L. Ryvarden	MH041538	MH041604
*S. meridense*	Costa Rica: Guanacaste	GB:KHL 11355	K.-H. Larsson	na	MH041583
*S. meridense*	Costa Rica: Guanacaste	GB:KHL 11365	K.-H. Larsson	MH041523	MH041584
*S. meridense*	Reunion: Saint-Benoit	LY 12816	G. Gilles	na	MH041597
*S. meridense*	Taiwan: Nantou	KAS:GEL 3520	E. Langer; G. Langer; C.-J. Chen	MH041548	na
S. aff. meridense	Argentina: Misiones	O:F:LR 19581	L. Ryvarden	MH041551	MH041578
S. aff. meridense	Brazil: Rondonia	O:F:KHL 15325	K.-H. Larsson	na	MH041585
S. aff. meridense	Colombia: Magdalena	O:F: 918846	L. Ryvarden	MH041554	MH041575
S. aff. meridense	Puerto Rico: Cerro Alto	GB:KHL 9561	K.-H. Larsson	MH041524	MH041581
S. aff. meridense	Puerto Rico: Luquillo	GB:KHL 10397	K.-H. Larsson	MH041519	MH041582
*S. nikau*	Reunion: Saint-Pierre	KAS:L 1296	J. Riebesehl; M. Schroth	MH041513	MH041565
*S. oberwinkleri*	Venezuela: Aragua	GB:KHL 11042	K.-H. Larsson	na	MH041561
*S. oberwinkleri* (holotype)	Reunion: Saint-Pierre	KAS:L 1860	J. Riebesehl	MH041511	MH041562
*S. obtusisporum*	Germany: Hesse	FR: Piepenbrink & Lotz-Winter W213-3-I	O. Koukol	MH041521	MH041566
*S. obtusisporum*	Jamaica: Cornwall	GB:KHL 10622	K.-H. Larsson	MH041559	MH041606
*S. parvisporum*	Reunion: Saint-Benoit	KAS:L 1226	J. Riebesehl	MH041528	MH041587
*S. parvisporum*	Reunion: Saint-Pierre	KAS:GEL 5032	E. Langer; E. Hennen	MH041530	MH041591
*S. parvisporum*	Reunion: Saint-Pierre	LY 12750	G. Gilles	na	MH041589
*S. parvisporum* (holotype)	Reunion: Saint-Pierre	KAS:L 0140	E. Langer	MH041529	MH041590
*S. perlongisporum*	Italy: Sicily	TU124388	A.Saitta	UDB028355*	UDB028355*
*S. perlongisporum*	Russia: Kaluga	LE 302156	S. Volobuev	KP268489*	na
*S. rarocrystallinum* (holotype)	Colombia: Cundinamarcha	O:F: 918488	L. Ryvarden	MH041512	MH041564
*S. robustius*	Jamaica: Cornwall	GB:KHL 10780	K.-H. Larsson	AY463468*	AY586714*
*S. robustius*	Puerto Rico: Luquillo	GB:KHL 10039	K.-H. Larsson	MH041515	na
*S. robustius*	Puerto Rico: Rio Grande	GB:KHL 10272	K.-H. Larsson	MH041516	MH041607
*S. robustius* (holotype)	Jamaica: Cornwall	GB:KHL 10813	K.-H. Larsson	MH041514	MH041608
*S. tedersooi*	Vietnam: Ninh Bình	TU 110895	L. Tedersoo	UDB014162*	na
S. tedersooi (holotype)	Vietnam: Ninh Bình	TU 110894	L. Tedersoo	UDB014161*	na
outgroup: *Brevicellicium exile*	Spain: Huesca	MA:F 26554	M. Dueñas,	HE963777*	HE963778*
outgroup: *Brevicellicium olivascens*	Sweden: Bohuslän	GB:KHL 8571	K.-H. Larsson	HE963792*	HE963793*

Sequences from each locus, ITS and 28S, were pre-aligned in Geneious version 5.6.7 (Kearse et al. 2012) with MUSCLE algorithm (eight iterations) (Edgar 2004). Final alignments of each locus were produced in the online mode of MAFFT version 7 (Katoh et al. 2017), with L-INS-i algorithm and other settings as default.

The small fragments of 18S rDNA and 28S rDNA were automatically trimmed from the target ITS region with the ITSx software (Bengtsson-Palme et al. 2013) implemented in the PlutoF workbench (Abarenkov et al. 2010). The same tool was used to partition ITS into ITS1, 5.8 and ITS2 regions prior to phylogenetic analyses of ITS alignment, to estimate the evolutionary model parameters for each partition separately. The 28S alignment was trimmed manually to produce the sequences of the same lengths and with fewer (if any) gaps at both ends and was not partitioned. Key properties of the final alignments were explored and described using RAxML terminology (Stamatakis 2016).

Morphologically outlined species were compared in terms of genetic distances estimated separately for the trimmed ITS and 28S alignments. For this, raw (also called uncorrected) pairwise dissimilarities of sequences in each alignment were calculated, defined as the percentage of sites that differ between each two full-length sequences including gap positions (Schoch et al. 2012, Kõljalg et al. 2013). This procedure was done with the “dist.dna” function of “ape” R package (Paradis et al. 2004) with option pairwise.deletion=FALSE (i.e. without deleting the sites with missing data in a pairwise way). Results were visualised with the ggplot2 R graphics (Wickham 2009) and R script can be viewed in Ordynets (2018a). Pairwise sequence dissimilarities were further analysed on the intraspecific versus interspecific level. The two levels of sequence variability were segregated with the “sppDist” function of the “spider” R package (Brown et al. 2012) and plotted simultaneously as histograms in a search for the barcoding gap (Meyer et al. 2005). As this classical approach provides only a general overview of sequence variability, i.e. for the pooled dataset, we applied also the recommended alternative which considers the species identity. For each sequence, the maximum intraspecific distance was contrasted with the minimum interspecific distance as recommended by Collins and Cruickshank (2012). Both types of distance for each sequence were estimated, respectively, with the functions “maxInDist” and “nonConDist” of the “spider” R package (Brown et al. 2012) and visualised as a scatterplot. R script for these procedures is available in Ordynets (2018c).

All phylogenetic analyses were performed using the GTR+G evolutionary model. We performed separate analyses of the ITS alignment (partitioned into ITS1, 5.8 and ITS2 regions), unpartitioned 28S alignment and concatenated ITS+28S alignment partitioned into four regions (ITS1, 5.8, ITS2 and 28S). For Bayesian inference of phylogeny, MrBayes 3.2.3 (Ronquist et al. 2012) was used. Two independent MCMC processes, each in 4 chains, were run. Five million trees were generated, sample frequency was set to 1000 and burnin fraction to 0.2. The acceptability of selected settings and mixing sampled trees, were confirmed by the standard deviation of split frequencies, by the potential scale reduction factor, by the sum of average effective sample size in two runs and by tracing likelihood scores of generated trees with Tracer 1.6 (Ronquist et al. 2011, Rambaut et al. 2014). For 8002 sampled trees per analysis (burn-in fraction excluded), a majority rule consensus tree was computed with branch supports representing the relative frequencies of bipartitions (posterior probabilities). Maximum likelihood analyses were performed in RAxML 8.2.10 (Stamatakis 2014). The search for the best-scoring maximum likelihood tree and bootstrap analysis (1000 replicates) were performed in a single run. Both RAxML and MrBayes were run on CIPRES Science Gateway V 3.3 (Miller et al. 2010; http://www.phylo.org). Resulting phylogenetic trees were first viewed in FigTree v. 1.4.2 (Rambaut 2014). Further visualisation and annotation of the phylogenetic trees were done in R version 3.3.3 (R Core Team 2017) and R script is available in Ordynets (2018d). The multiple sequence alignments, details of phylogenetic analyses and trees generated in the study were deposited in TreeBASE: http://purl.org/phylo/treebase/phylows/study/TB2:S22473.

## Results

### Descriptions of new species

#### Subulicystidium boidinii

Taxon classificationFungiTrechisporalesHydnodontaceae

Ordynets, M.M.Striegel & Langer sp. nov.

Figs 5e–g ; 10q

##### Diagnosis.

Species with broader allantoid spores (2.8–3.5 µm) and less heavily encrusted cystidia than in *Subulicystidium meridense* Oberw.

##### Type.

RÉUNION. Saint-Benoît: Salazie, Hell Bourg, ca 1000 m, -21.0642, 55.5269, on dead woody branch, 23 Mar 2015, M.Striegel (L 1584a in FR; isotype in KAS).

##### Etymology.

*boidinii*, in honour of Jacques Boidin, a great explorer of fungi of Réunion Island, who collected this species and suggested an independent status for it.

##### Description.

*Basidiomata* annual, effused, resupinate, soft and fragile, arachnoid, thin, loosely adnate. Hymenophore smooth, finely velutinous due to numerous protruding cystidia, whitish. Margin thinning out, pruinose, adnate.

*Hyphal system* monomitic. All septa with clamps. Subiculum thin, with loosely interwoven richly branched hyphae 1.5–2.5 µm wide, thin-walled, hyaline and smooth. Subhymenium thin, with hyphae similar to those in subiculum but occasionally bearing slight amorphous hyaline encrustation. *Cystidia* subulate, rather narrow, 45–65 × 2.5–3.5 µm including encrustation, projecting up to 30 µm, without basal swelling, terminal or pleural, with thin hyaline cell wall and outer hyaline crystalline sheath covering the whole cystidia except the tapering, thin-walled, acuminate apex. Crystal protrusions on cystidium are small and clearly rectangular and arranged in longitudinal rows.

*Basidia* suburniform to almost clavate, 10–12 × 4–5.5 µm, thin-walled, with 4 sterigmata and a basal clamp, without or with slight amorphous hyaline encrustation at the base. *Basidiospores* allantoid, L=(5.7–)5.9–7.2(–7.9) µm, W=(2.6–)2.8–3.3(–3.5) μm, Q=(1.8–)2.0–2.4(–2.5), N=116/2, with minute apiculus, smooth, thin-walled, hyaline, often with two oil drops (one at each pole), negative in Melzer’s reagent.

##### Additional specimens examined.

COSTA RICA. Puntarenas: Coto Brus, Sabalito, Zona Protectora Las Tablas, Finca Cafrosa, El Tajo, 1560 m, 8.9225, -82.7956, on stem of angiosperm tree, 6 Nov 2004, K.-H.Larsson (KHL 12830 in GB). RÉUNION. Saint Pierre: Cilaos, A_Cilaos X, Forêt de la Mare-a-Joseph, kiosque au milieus des *Cryptomeria* D.Don, alt. 1400 m, on strongly decayed wood of *Cryptomeria japonica* D.Don, 20 Apr 1985, J.Boidin (LY 11247).

##### Remarks on species.

Boidin and Gilles (1988) in their survey of *Subulicystidium* from Réunion Island reported “*Subulicystidium allantosporum* ad interim” and referred to their specimens LY 11247 and LY 12750. We show in the current study that these two collections represent different species and only the former may be assigned to *S. boidinii*. We were not able to sequence LY 11247, but description and illustration (fig. 38A) provided by Boidin and Gilles (1988) and our re-measuring of basidiospores in their specimen (Supplementary files 2 and 3) agree well with our concept of *S. boidinii*.

#### Subulicystidium fusisporum

Taxon classificationFungiTrechisporalesHydnodontaceae

Ordynets & K.H.Larss. sp. nov.

Figs 4a–c ; 10e

##### Diagnosis.

Differs from *Subulicystidium longisporum* (Pat.) Parmasto by fusiform basidiospores which are ca. 10–13 µm long and 2.5–3.5 µm broad.

##### Type.

PUERTO RICO. Municipio Rio Grande, Luquillo Mts, El Verde Research Area, between Field Station and 16-hectare grid, 320–380 m, 18.3233, -65.8172, on fallen tree log, 9 Jun 1998, K.-H.Larsson (KHL 10360 in GB).

##### Etymology.

*fusisporum* (Lat.), having fusiform basidiospores.

##### Description.

*Basidiomata* annual, effused, resupinate, soft and fragile, arachnoid, thin, loosely adnate. Hymenophore smooth, finely velutinous due to numerous protruding cystidia, whitish. Margin thinning out, adnate.

*system* monomitic. All septa with clamps. Subiculum thin, with loosely interwoven richly branched hyphae 2.5–3.5 µm wide, usually thin-walled, hyaline and smooth. Subhymenium thin, with hyphae slightly broader than in subiculum, 2.7–4 µm wide, compactly arranged, often slightly thick-walled and covered with hyaline crystalline sheath. *Cystidia* subulate, 65–90 × 3.5–5 µm including encrustation, projecting up to 40 µm, without or occasionally with basal swelling (up to 6 µm wide), terminal, with thick hyaline cell wall and outer hyaline crystalline sheath covering the whole cystidium except the thin-walled, acuminate apex. Crystal protrusions on cystidium are small to moderately large and clearly rectangular and arranged in longitudinal rows.

*Basidia* suburniform, 12–14 × 4.5–6 µm, thin-walled, with 4 sterigmata and a basal clamp, often with a hyaline crystal collar at the base. *Basidiospores* fusiform, L=(9.7–)10.7–12.8(–13.3) µm, W=(2.1–)2.4–3.4(–3.7) µm, Q= (3.0–)3.3–4.9(–5.9), N=127/3, with minute apiculus, smooth, thin-walled, hyaline, occasionally with oil drops, negative in Melzer’s reagent. Tolerance limits for basidiospore length, width and length to width ratio in *S. fusisporum* based on 3 sequenced specimens are provided in the Table 2.

**Table 2.** 90% tolerance limits defined for the 90% probability level for the mean basidiospore length, width and length to width ratio for *Subulicystidium* species with at least 3 sequenced specimens. The following specimens were used to estimate tolerance limits for species: *Subulicystidium fusisporum*: GB:KHL 9093, 10360 and 12761; *S. harpagum*: GB:KHL 10733, O:F:LR 15736, KAS:L 0244 and 1726a; *S. parvisporum*: KAS:GEL 5032, KAS:L 0140 and 1226 and LY 12750; *S. robustius*: GB:KHL 10039, 10272, 10780 and 10813.

Measurement type	Estimate	Species			
		*Subulicystidium fusisporum*	*Subulicystidium harpagum*	*Subulicystidium parvisporum*	*Subulicystidium robustius*
Spore length, µm	Sample mean	11.78	6.74	5.61	9.78
	Lower limit of 90% tolerance interval	9.64	4.34	4.78	7.81
	Upper limit of 90% tolerance interval	13.92	9.13	6.43	11.75
Spore width, µm	Sample mean	2.92	2.6	2.51	3.00
	Lower limit of 90% tolerance interval	1.65	1.62	2.06	2.44
	Upper limit of 90% tolerance interval	4.19	3.58	2.95	3.57
Spore length/width ratio	Sample mean	4.09	2.63	2.25	3.27
	Lower limit of 90% tolerance interval	1.89	0.83	1.91	2.52
	Upper limit of 90% tolerance interval	6.28	4.42	2.59	4.02

##### Additional specimens examined.

COSTA RICA. Puntarenas: Coto Brus, Sabalito, Zona Protectora Las Tablas, La Neblina, 8.9149, -82.7719, on stem of angiosperm tree, 5 Nov 2004, K.-H.Larsson (KHL 12761 in GB). CÔTE D’IVOIRE. Abidjan: Foret du Banco, 5.3932, -4.0525, on dead wood, 6 Jul 1974, G.Gilles (LY 7375). JAMAICA. Cornwall County: Trelawny parish, N of Crowlands, trail/road into park area, 18.2611, -77.6511, on stem of angiosperm tree, 10 Jun 1999, K.-H.Larsson (KHL 10612 in GB). PUERTO RICO. Municipio Rio Grande, Luquillo Mts, El Verde Research Area, between Field Station and 16-hectare grid, 320-380 m, 18.3233, -65.8172, on strongly decayed stem of angiosperm tree, 19 Jun 1996, K.-H.Larsson (KHL 9093 in GB), on uprooted angiosperm tree, 19 Jun 1996, K.-H.Larsson (KHL 9061 in GB).

##### Remarks on species.

Amongst the species considered in this study, *S. fusisporum* is the most probable to be confused with *S. longisporum.* However, careful measurement of basidiospores (length below 13 µm, see Fig. 10e vs. 10g) and rDNA sequence identity clearly point to the species of its own. The regular rectangular shape of crystal protrusion as well as their dense arrangement in longitudinal rows on cystidia in *S. fusisporum* is also prominent.

#### Subulicystidium grandisporum

Taxon classificationFungiTrechisporalesHydnodontaceae

Ordynets & K.H.Larss. sp. nov.

Figs 6a–c ; 10i

##### Diagnosis.

Species with the largest cylindrical basidiospores ever observed in the genus (10.5–14.5 × 3.3–3.9 µm) and relatively large cystidia with prominent regular encrustation.

##### Type.

COSTA RICA. Cartago: Faldas del volcano Irazu, 1800 m, on decayed twig, 28 May 1991, L.Ryvarden (LR 29162 in O:F 506781).

##### Etymology.

*grandisporum* (Lat.), having large basidiospores.

##### Description.

*Basidiomata* annual, effused, resupinate, soft and fragile, arachnoid, thin, loosely adnate. Hymenophore smooth, finely velutinous due to numerous protruding cystidia, whitish. Margin thinning out, pruinose, adnate.

*system* monomitic. All septa with clamps. Subiculum thin, with loosely interwoven richly branched hyphae 3–4 µm wide, thin-walled, hyaline and smooth. Subhymenium thin, compact, with richly branched hyphae 3–3.5 µm wide, often covered with thin hyaline crystalline sheath. *Cystidia* subulate, 70–90 × 5–7 µm including encrustation, projecting up to 60 µm, without basal swelling, terminal or pleural, with thick hyaline cell wall and outer hyaline crystalline sheath covering the whole cystidium except the thin-walled, tapering apex. Crystal protrusions on cystidium are large, clearly rectangular to rounded, rather sparsely arranged in longitudinal rows.

*Basidia* suburniform, 13–19 × 5.5–7 µm, thin-walled, with 4 sterigmata and a basal clamp, often with hyaline crystalline collar at the base. *Basidiospores* cylindric, adaxial side slightly concave, L=(10–)10.6–14.5(–15.3) µm, W=(3.2–)3.3–3.9(–4.2) µm, Q= (2.9–)3.0–4.0, N=48/1, with minute apiculus, smooth, thin-walled, hyaline, negative in Melzer’s reagent.

*Remarks on species*. Until now, it is the only known *Subulicystidium* species with such large cylindrical basidiospores. Additionally, large cystidia with regular large protrusions, together with large basidia, make the species remarkable.

#### Subulicystidium harpagum

Taxon classificationFungiTrechisporalesHydnodontaceae

Ordynets, M.M.Striegel & K.H.Larss. sp. nov.

Figs 7a, b ; 10r

##### Diagnosis.

Differs from other *Subulicystidium* species by the cystidia which resemble a harpoon due to protruded backward pointing individual crystals and moderately large cylindric to allantoid basidiospores (5.7–8.2 × 2.2–3.0 µm).

##### Type.

RÉUNION. Saint-Pierre: Saint-Philippe, Forêt de Mare Longue, 495 m, -21.3438, 55.7410, on dead tree branch, 28 Mar 2015, M.Striegel (L 1726a in FR, isotype in KAS).

##### Etymology.

*harpagum*, from the Latin “harpaga”, English “harpoon”, a spear with barbs and serrated edges used in fishing. Epithet refers to the cystidium encrustation pattern.

##### Description.

*Basidiomata* annual, effused, resupinate, soft and fragile, arachnoid, loosely adnate and easily separable. Hymenophore smooth, velutinous due to numerous protruding large cystidia, whitish. Margin not differentiated.

*system* monomitic. All septa with clamps. Subiculum thin, with interwoven richly branched hyphae 2-3 µm wide, thin-walled to very slightly thick-walled, hyaline, often with rough surface because of slight encrustation. In the older fruit-body parts, encrustation represents an up to 1 µm thick sheath over the hypha. Subhymenium thin, with hyphae identical to those in subiculum. *Cystidia* subulate, 35–62 × 2.5–3.5 µm including encrustation, projecting up to 30 µm, without basal swelling, terminal or pleural, with thin to slightly thickened hyaline cell wall and outer hyaline crystal sheath covering the whole cystidium except the thin-walled, acuminate and particularly narrow, apex. Crystal protrusions on cystidium are formed like short rods that project backwards under acute angle, thus making cystidia resembling a harpoon.

*Basidia* suburniform, 9–12 × 4.2–5.7 µm, thin-walled, with 4 sterigmata and a basal clamp, basally slightly encrusted. *Basidiospores* weakly allantoid, adaxial side concave, L=(4.5–)5.7–8.2(–8.7) µm, W=(2.0–)2.2–3.0(–3.3) µm, Q=(1.7–)2.1–3.4(–3.8), N=178/4, with minute apiculus, smooth, thin-walled, hyaline, often with two oil drops (one at each pole), negative in Melzer’s reagent. Tolerance limits for basidiospore length, width and length to width ratio in *S. harpagum* based on 4 sequenced specimens are provided in Table 2.

##### Additional specimens examined.

RÉUNION. Saint-Benoît: Sainte-Rose, Forêt de Bois Blanc, 640 m, -21.2081, 55.7981, on strongly decayed wood, 21 Mar 2013, E.Langer (L 0244 in FR and KAS). JAMAICA. Cornwall County: Trelawny parish, Windsor Cave, along trail to Troy, 18.3564, -77.6472, on twig of angiosperm tree, 12 Jun 1999, K.-H.Larsson (KHL 10733 in GB). COLOMBIA. Magdalena: Parque Nacional Tayrona, Estacion de Gairaca, 0-30 m, 11.3170, -74.1063, on dead twig, 12 Jun 1978, L.Ryvarden (LR 15736 in O:F).

##### Remarks on species.

The holotype specimen contains also a small piece of *S. perlongisporum*, now kept in a separate clearly labelled envelope within the voucher. Despite being mixed, the specimen was still selected as type because of the hymenium and subhymenium are better preserved and the ITS and 28S sequences retrieved are of higher quality.

#### Subulicystidium inornatum

Taxon classificationFungiTrechisporalesHydnodontaceae

Ordynets & K.H.Larss. sp. nov.

Figs 4d–f ; 10d

##### Diagnosis.

The species has cystidia that do not possess individual crystal protrusions but are instead smooth or only slightly rough and basidiospores that are fusiform and moderately large, 8.1–10.9 × 2.7–3.3 µm.

##### Type.

PUERTO RICO. Municipio Rio Grande, Luquillo Mts, El Yunque, Mount Britton Trail, between upper road and trail head, 760-880 m, 18.3003, -65.7917, on wet dead wood, 11 Jun 1998, K.-H.Larsson (KHL 10444 in GB).

##### Etymology.

*inornatum* (Lat.), without ornament, referring to the almost smooth cystidia.

##### Description.

*Basidiomata* annual, effused, resupinate, soft and fragile, arachnoid, thin, loosely adnate. Hymenophore smooth, finely velutinous due to numerous protruding cystidia, whitish. Margin thinning out, adnate.

*system* monomitic. All septa with clamps. Subiculum thin, with loosely interwoven richly branched hyphae 3–4 µm wide, hyaline, thin-walled to slightly thick-walled, covered by a thin hyaline crystal sheath giving them a slightly rough appearance. Subhymenial hyphae similar to those in subiculum, but more compactly arranged and slightly agglutinated. *Cystidia* subulate, 45–60 × 4–5.5 µm including encrustation, projecting up to 45 µm, occasionally with slight basal swelling (up to 6 µm), terminal, thick-walled and with an outer hyaline crystal sheath covering the whole cystidium except the thin-walled acuminate apex. Surface of the crystal sheath slightly rough, crystal protrusions lacking.

*Basidia* suburniform to almost clavate, 10–14 × 4.5–6 µm, thin-walled, with 4 sterigmata and a basal clamp, often with hyaline crystalline collar at the base. *Basidiospores* fusiform, L=(7.2)8.1–10.9(–11.0) µm, W=(2.5–)2.7–3.3(–3.5) µm, Q=(2.4–)2.7–3.8(–4.1), N=97/1, with minute apiculus, smooth, thin-walled, hyaline, negative in Melzer’s reagent.

##### Additional specimens examined.

COSTA RICA. Puntarenas: Carrara Biologica Reserva, ca. 50 m, 9.7472, -84.6278, on dead fruit-bodies of *Coriolopsis rigida* (Berk. & Mont.) Murill, 14 Jun 1991, L.Ryvarden (LR 29823 in O:F 506780). PUERTO RICO. Municipio Cayey, Bosque Estatal Carite, Guavate Picnic area, 18.1264, -66.0764, on dead wood, 23 Jun 1996, K.-H.Larsson (KHL 9289 and 9337 in GB).

##### Remarks on species.

This is the only species in which cystidia and hyphae have a similar surface, which is smooth or slightly rough due to a thin layer of crystalline matter.

#### Subulicystidium oberwinkleri

Taxon classificationFungiTrechisporalesHydnodontaceae

Ordynets, Riebesehl & K.H.Larss. sp. nov.

Figs 5a, b ; 10t

##### Diagnosis.

differs from *Subulicystidium nikau* (G. Cunn.) Jülich by having plate-like to irregular crystals on cystidium and longer basidiospores (7.8–10.8 µm long).

##### Type.

RÉUNION. Saint-Pierre: Saint-Philippe, Forêt de Mare Longue, 495 m, -21.3438, 55.7410, on dead woody branch, 28 Mar 2015, J.Riebesehl (L 1860 in FR; isotype in KAS).

##### Etymology.

*oberwinkleri*, named after Franz Oberwinkler, a German mycologist who provided a perceptive view into the species concepts in *Subulicystidium* and was an early collector of the species in South America.

##### Description.

*Basidiomata* annual, effused, resupinate, soft and fragile, arachnoid, loosely adnate and easily separable. Hymenophore smooth, velutinous due to numerous protruding large cystidia, porulose, whitish to yellow. Margin abrupt, not differentiated.

*system* monomitic. All septa with clamps. Subiculum with interwoven and richly branched hyphae 3–4 µm wide, occasionally swollen up to 6 µm, slightly to moderately thick-walled, hyaline. Subhymenium thin and loose. Subhymenial hyphae richly branched, intricate, regular or occasionally slightly inflated, 3–4 µm wide, thin-walled. *Cystidia* tubular, 80–150 × 5.5–10 µm including encrustation, projecting up to 70 µm, without basal swelling, with septa having or devoid of clamps, with thin or only slightly thickened hyaline cell wall and outer hyaline crystalline sheath (up to 3.5 µm thick) covering at least the lower half and, at a maximum, almost the whole cystidium except the short, 2–3 µm wide, hyphoid, cylindrical or tapering apex. The crystal protrusions on cystidium are large, plate-like, slightly rhomboid or irregular in outline, somewhat imbricately arranged. Similar encrustation pattern is found also on the subicular and especially subhymenial hyphae and sometimes on the bases of basidia.

*Basidia* suburniform to urniform, 12–18 × 6–8 µm, thin-walled, with 4 sterigmata and a basal clamp, terminal or sometimes pleural. *Basidiospores* broad cylindric to reniform, adaxial side slightly concave, L=(7.4–)7.8–10.8(–11.6) µm, W=(3.7–)4.0–5.5(–5.8) µm, Q=(1.6–)1.6–2.3(–2.4), N=99/3, with a prominent apiculus, smooth, thin-walled, hyaline, negative in Melzer’s reagent.

##### Additional specimens examined.

RÉUNION. Saint-Benoit: Saint-Benoit, Forêt de Bébour, Bebour-I-87, *Cryptomeria* forest, 1200 m, on dead wood of *Cryptomeria japonica*, 24 May 1987, J.Boidin (LY 12488). VENEZUELA. Estado Aragua: Maracay, National Park Henri Pittier, Rancho Grande, 10.3800, -67.6190, on dead wood, 30 Aug 1999, K.-H.Larsson (KHL 11042 in GB). Estado Merida: La Carbonera, Road Merida-La Azulita, 2000–2200 m, on dead wood, 19 Jan 1969, F.Oberwinkler (FO 14338 in TUB).

##### Remarks on species.

Specimens of *S. oberwinkleri* were noticed for the peculiar cystidia previously by Oberwinkler (1977) and later by Maekawa (1998). Neither author was prepared to assign them to a separate species and instead labelled them as *S. nikau* (characterised by regularly ornamented cystidia). Our examination of the specimens TUB:FO 14338 from Venezuela (Oberwinkler 1977, fig. 31) and LY 12488 from Réunion (Boidin and Gilles 1988, fig. 39A) showed that both represent *S. oberwinkleri*.

#### Subulicystidium parvisporum

Taxon classificationFungiTrechisporalesHydnodontaceae

Ordynets & Langer sp. nov.

Figs 7c, d ; 10s

##### Diagnosis.

The species with the smallest basidiospores known in the genus, 5.0–6.2 × 2.2–2.8 µm and allantoid, combined with rather small cystidia with regular delicate encrustation.

##### Holotype.

RÉUNION. Saint-Pierre: Cilaos, Cirque de Cilaos, Roche Merveilleux, Sentiere botanique, 1300 m, -21.1232, 55.4920, on strongly decayed wood, 15 Mar 2013, E.Langer (L 0140 in FR; isotype in KAS).

##### Etymology.

*parvisporum* (Lat.), having small basidiospores.

##### Description.

*Basidiomata* annual, effused, resupinate, soft and fragile, arachnoid, thin, loosely adnate. Hymenophore smooth, finely velutinous due to numerous protruding cystidia, whitish. Margin thinning out, pruinose, adnate.

*system* monomitic. All septa with clamps. Subiculum thin, with loosely interwoven richly branched hyphae 1.8–3 µm wide, thin-walled, hyaline and smooth. Subhymenium thin, with hyphae similar to those in subiculum but occasionally bearing slight amorphous hyaline encrustation. *Cystidia* subulate, 45–65 × 2.5–3 µm including encrustation, projecting up to 30 µm, without basal swelling, terminal or pleural, with thin hyaline cell wall and outer hyaline crystalline sheath covering the whole cystidium except the thin-walled, narrow, acuminate apex. Crystal protrusions on cystidium are low but clearly rectangular and arranged in longitudinal rows.

*Basidia* suburniform to almost clavate, 10–15 × 4–5 µm, thin-walled, with 4 sterigmata and a basal clamp, occasionally with slight amorphous hyaline encrustation at the base. *Basidiospores* allantoid, often with a slight constriction in the middle part, L= (4.3)5.0–6.2(–6.8) µm, W=(1.8–)2.2–2.8(–3.0) µm, Q=(1.8–)1.9–2.6(–3.1), N=151/4, with minute apiculus, smooth, thin-walled, hyaline, occasionally with one or two oil drops, negative in Melzer’s reagent. Tolerance limits for basidiospore length, width and length to width ratio in *S. parvisporum*, based on 4 sequenced specimens, are provided in the Table 2.

##### Additional specimens examined.

RÉUNION. Saint-Benoit: Saint-Benoit, Forêt Margarithe, ca. 450 m, -21.1031, 55.6926, on dead wood, 24 Mar 2015, J.Riebesehl (L 1226 in FR and KAS). Saint-Pierre: Cilaos, Cilaos XII-87, forêt de la Mare à Joseph, au-dessus du hameau de Bras Sec, 1400 m, -21.1239, 55.4957, on dead wood, 4 Apr 1987, G.Gilles (LY 12750); le Tampon, Notre dame de la Paix, Forêt de la Riviere des Remparts, Sentier Botanique, -21.2559, 55.5987, on dead wood, 23 Mar 1998, E.Langer & E.Hennen (GEL 5032 in KAS).

##### Remarks on species.

Boidin and Gilles (1988) mentioned one collection with such small spores for his *S. allantosporum* ad interim (LY12750). After examining and sequencing the specimen, we conclude that it clearly represents our new species *S. parvisporum*. Both ours and specimens of Boidin and Gilles originate exclusively from Réunion.

#### Subulicystidium rarocrystallinum

Taxon classificationFungiTrechisporalesHydnodontaceae

Ordynets & K.H.Larss. sp. nov.

Figs 6f–h ; 10k

##### Diagnosis.

Differs from all other *Subulicystidium* species by cystidia which bear few spaced and irregularly located crystals and have a thick cell wall.

##### Type.

COLOMBIA. Cundinamarcha: 23rd kilometre of a highway from Medellin (direction SE) to Tenjo, alt 2600 m, 6.0605, -75.4095, on dead twig, 4 Jun 1978, L.Ryvarden (LR 15483 in O:F 918488).

##### Etymology.

*rarocrystallinum* (Lat.), having few spaced crystals on cystidium.

##### Description.

*Basidiomata* annual, effused, resupinate, fragile, porulose, thin, adnate. Hymenophore smooth, finely velutinous due to numerous protruding cystidia, whitish. Margin thinning out, adnate.

*system* monomitic. All septa with clamps. Subiculum thin, compact, with richly branched hyphae 3–3.5 µm wide, thin-walled to slightly thick-walled, hyaline and smooth. Subhymenium thin, compact, with richly branched hyphae 3–3.5 µm wide, thin-walled, smooth. *Cystidia* subulate, 45–65(–80) × 3.7–5 µm including encrustation, projecting up to 50 µm, with especially thick-walled, occasionally slightly swollen (up to 5.5 µm), basal part, with outer hyaline crystalline sheath covering the whole cystidium except the tapering, thin-walled apex. Crystal protrusions on cystidium are moderately large, rectangular to rounded, rather sparse and allocated rather irregularly and mostly in the medial part.

*Basidia* suburniform, 11–15 × 4.5–5.5 µm, thin-walled, with 4 sterigmata and a basal clamp, without encrustation. *Basidiospores* cylindric, adaxial side slightly concave, L=(7.5–)8.0–10.5(–10.8) µm, W=(2.8)2.9–3.7(–3.8) µm, Q=(2.2–)2.5–3.2(–3.7), N=72/1, with minute apiculus, smooth, thin-walled, hyaline, negative in Melzer’s reagent.

##### Remarks on species.

The few spaced far from each other crystals on cystidium and thick cell wall of cystidium are peculiar. Furthermore, in the single collection studied, cystidia were relatively infrequent and subhymenium was more compact than in other species. Species can be distinguished from *Subulicystidium brachysporum* also by larger cylindric basidiospores.

#### Subulicystidium robustius

Taxon classificationFungiTrechisporalesHydnodontaceae

K.H.Larss. & Ordynets sp. nov.

Figs 3f–h ; 10c

##### Diagnosis.

The species is characterised by numerous large and most prominently ornamented cystidia with regular ornamentation and by moderately broad fusiform basidiospores 10.5–12.5 × 2.5–3.5 µm.

##### Type.

JAMAICA. Cornwall County: Trelawny parish, Windsor Cave, along trail to Troy, 18.3564, -77.6472, on trunk of angiosperm tree, 13 Jun 1999, K.-H.Larsson (KHL 10813 in GB).

##### Etymology.

*robustius* (Lat.), having large cystidia with large crystal protrusions.

##### Description.

*Basidiomata* annual, effused, resupinate, soft and fragile, arachnoid, loosely adnate and easily separable. Hymenophore smooth, hirsute due to numerous protruding large cystidia, yellowish. Margin thinning out, pruinose, adnate.

*system* monomitic. All septa with clamps. Subiculum thick, with interwoven richly branched hyphae 2–3 µm wide, thin-walled to slightly thick-walled, hyaline to yellowish, smooth or with sparse granulose encrustation. Subhymenium rather thick, up to 60 µm. Subhymenial hyphae richly branched, intricate, regular or occasionally slightly inflated, 2–4 µm wide, thin-walled, occasionally weakly encrusted by yellowish crystalline material. *Cystidia* subulate, 80–105 × 4.5–6 µm including encrustation, projecting up to 65 µm, without basal swelling, terminal or pleural, with thick yellowish wall and outer hyaline crystalline sheath covering the whole cystidium except the small tapering or acuminate apex. Crystal protrusions on cystidium are large and clearly rectangular, arranged in longitudinal rows.

*Basidia* clavate to suburniform, 13–20 × 4–6 µm, thin-walled, with 4 sterigmata and a basal clamp, without encrustation or rarely with a slight crystalline crust at the base. *Basidiospores* fusiform, adaxial side convex, L= (8.1–)8.5–10.9(–11.7) µm, W=(2.5–)2.7–3.5(–3.7) µm, Q=(2.4–)2.6–3.6(–4.0), N=197/4, with minute apiculus, smooth, thin-walled, hyaline, negative in Melzer’s reagent. Tolerance limits for basidiospore length, width and length to width ratio in *S. robustius*, based on 4 sequenced specimens, are provided in Table 2.

##### Additional specimens examined.

BRAZIL. Sao Paulo: Cananeia, Ilha do Cardoso, -25.1336, -47.9617, on dead wood, 2-5 Feb 1987, D.Pegler, K.Hjortstam & L.Ryvarden (LR 24792 in O:F). COLOMBIA. Magdalena: Parque Nacional Tayrona, Estacion de Gairaca, 0-30 m, 11.3170, -74.1063, on dead wood, 12 Jun 1978, L.Ryvarden (LR 15791 in O:F 918494). COSTA RICA. Alajuela: Bijagua, Albergue Heliconias, Sendero Heliconias, 770 m, 10.7181, -85.0453, on log of angiosperm tree, 12 Jul 2001, K.-H.Larsson (KHL 11245 and 11259 in GB); San Ramon, Reserva Forestal Colonia Palmarena, 850 m., 10.2500, -84.5667, on dead wood, 14 Mar 1991, L.Horovitz (FO 42968 in TUB). ECUADOR. Orellana: Yasuni National Park, Yasuni Scientific Research Station, -0.6859, -76.3953, on dead wood, 9-12 Mar 2002, L.Ryvarden (LR 44667 in O:F 505981 and LR 44688 in O:F 505799). JAMAICA. Cornwall County: Trelawny parish, N of Crowlands, trail/road into park area, 18.2611, -77.6511, on stem of angiosperm tree, 10 Jun 1999, K.-H.Larsson (KHL 10661 in GB); Windsor Cave, along trail to Troy, 18.3564, -77.6472, on trunk of angiosperm tree, 13 Jun 1999, K.-H.Larsson (KHL 10780 and 10814 in GB). Surrey County: Portland parish, between reach and Ecclesdown hillside to the east, alt 500 m, 18.0433, -76.3108, on of angiosperm trunk, 16 Jun 1999, K.-H.Larsson (KHL 10895 in GB). PUERTO RICO. Municipio Juana Diaz, Bosque Estatal Toro Negro, near DNR office, downstream from Road 143, 18.1539, -66.5356, on dead wood, 24 Jun 1996, K.-H.Larsson (KHL 9381 in GB). Municipio Luquillo, Luquillo Mts, Bisley Experimental Watersheds, along the logging road, 215 m a.s.l., 18.3161, -65.7467, on log of angiosperm tree, 6 Jun 1997, K.-H.Larsson (KHL 10039 in GB); Sabana, above Chicken Farm & Rio Sabana, 70 m a.s.l., 18.3500, -65.7344, on log, 10 Jun 1998, K.-H.Larsson (KHL 10423 in GB). Municipio Maricao, Reserva Forestal Maricao, near Fish Hatchery, 18.1922, -66.9933, on decaying log of angiosperm tree, 25 Jun 1996, K.-H.Larsson (KHL 9454 in GB). Municipio Rio Grande, Luquillo Mts, El Verde Research Area, between Field Station and 16-hectare grid, 320-380 m, 18.3233, -65.8172, on log, 7 Jun 1998, K.-H.Larsson (KHL 10272 in GB); El Verde Research Area, lower part of 16-hectare grid, 345-360 m, 18.3239, -65.8172, on dead wood, 28 Jun 1996, K.-H.Larsson (KHL 9574 in GB). VENEZUELA. Estado Amazonas: Manapiare, Yutajé, 5.6142, -66.1236, on dead wood of angiosperm tree, 12-19 Apr 1998, L.Ryvarden (LR 40545 in O:F). Estado Aragua: Maracay, National Park Henri Pittier, Rancho Grande, 10.3800, -67.6190, on dead wood of angiosperm tree, 25 Apr 1998, L.Ryvarden (LR 40767 in O:F).

##### Remarks on species.

Our data shows that the species is widespread in the Caribbean region and in South America. We were able to examine the specimen mentioned and illustrated from Costa Rica by Kisimova-Horovitz et al. (1997) under the name *S. naviculatum* and re-identified it as *S. robustius*.

#### Subulicystidium ryvardenii

Taxon classificationFungiTrechisporalesHydnodontaceae

Ordynets & K.H.Larss. sp. nov.

Figs 3c–e ; 10b

##### Diagnosis.

Species with fusiform basidiospores with the width range 3.5–4.2 µm, halfway between the width ranges of *Subulicystidium robustius* K.H. Larss. & Ordynets and *S. naviculatum* Oberw.

##### Type.

ETHIOPIA. Arussi: Munessa Forest east of Lake Lagano, 7.5833, 38.9167, on dead wood, 10 Jan 1973, L.Ryvarden (LR 8860/b in O:F 909583).

##### Etymology.

*ryvardenii*, named after Leif Ryvarden, a Norwegian mycologist, enthusiastic explorer of the tropical fungal diversity and collector of the type specimen.

##### Description.

*Basidiomata* annual, effused, resupinate, soft and fragile, arachnoid, thin, loosely adnate. Hymenophore smooth, hirsute due to numerous large protruding cystidia, yellowish. Margin thinning out, adnate.

*system* monomitic. All septa with clamps. Subiculum thin, with loosely interwoven richly branched hyphae 3–4 µm wide, thin-walled to slightly thick-walled, hyaline and smooth. Subhymenium weakly developed, with hyphae 3–4 µm wide, loosely arranged, slightly thick-walled and often covered with a hyaline crystal sheath. *Cystidia* subulate, 65–115 × 4.5–6 µm including encrustation, projecting up to 50 µm, with or without a slight basal swelling (up to 6.5 µm diam.), terminal, with thick hyaline cell wall and an outer hyaline crystal sheath covering the whole cystidium except the tapering, thin-walled apex. Crystal protrusions on cystidium are large and mostly rounded and sparsely arranged in longitudinal rows.

*Basidia* subclavate to suburniform, 15–20 × 4–5 µm, thin-walled, with 4 sterigmata and a basal clamp, often with a hyaline crystal collar at the base. *Basidiospores* broadly fusiform, L=(8.5–)8.7–11.2(–11.6) µm, W= 3.5–4.2(–4.4) µm, Q=(2.3–)2.4–3.0(–3.2), N=31/1, with minute apiculus, smooth, thin-walled, hyaline, occasionally with oil drops, negative in Melzer’s reagent.

##### Remarks on species.

With its hirsute hymenium which has numerous large cystidia, the species is similar to *S. robustius*, but differs by broader basidiospores and more rounded single crystals on cystidia.

#### Subulicystidium tedersooi

Taxon classificationFungiTrechisporalesHydnodontaceae

Ordynets, Scherf & Langer sp. nov.

Figs 4g, h ; 10f

##### Diagnosis.

Species with particularly narrow fusiform basidiospores, 8.5–11.5 × 2–2.5 µm and long, 85–125 µm, regularly encrusted cystidia.

##### Type.

VIETNAM. Ninh Bình Province: Cuc Phuong National Park, sampling area G2906, 20.3500, 105.6026, on fallen decayed twig, 15 Oct 2012, L.Tedersoo (TU 110894).

##### Etymology.

*tedersooi*, named after Leho Tedersoo, an Estonian mycologist, the vigorous explorer of the global soil fungal diversity and collector of the type specimen.

##### Description.

*Basidiomata* annual, effused, resupinate, soft and fragile, arachnoid, thin, loosely adnate. Hymenophore smooth, finely velutinous due to numerous protruding cystidia, whitish. Margin thinning out, adnate.

*system* monomitic. All septa with clamps. Subicular and subhymenial layer weakly differentiated, consisting of richly branched hyphae 2–3 µm wide, thin-walled, with rough surface due to a subinvisible hyaline crystal sheath. *Cystidia* subulate, 85–125 × 4.5–5 µm, usually without basal swelling, terminal, with thick hyaline cell wall and an outer hyaline crystal sheath covering the whole cystidium except the acuminate apex. Crystal protrusions on cystidium are rectangular, moderately large, regularly arranged in longitudinal rows.

*Basidia* suburniform to cylindrical, 9–13 × 4.5–5, thin-walled, with 4 sterigmata and a basal clamp, occasionally with a thin hyaline crystal collar at the base. *Basidiospores* narrowly fusiform, L=(7.9–)8.4–11.5(–11.8) µm, W=(1.9–)2.1–2.6(–2.8) µm, Q=(3.4–)3.5–5.0(–5.7), N=81/2, with straight to slightly curved base, thin-walled, often with two large or many smaller oil drops, negative in Melzer’s reagent.

##### Additional specimens examined.

VIETNAM. Ninh Bình Province: Cuc Phuong National Park, sampling area G2906, 20.3500, 105.6026, on fallen decayed twig, 15 Oct 2012, L.Tedersoo (TU 110895).

##### Remarks on species.

The narrow spores of *S. tedersooi* are comparable in width only with *S. perlongisporum* (see Fig. 10f vs. 10j). However, the spore length of two species drastically differs: 8.4–11.5 µm in *S. tedersooi* vs. 17–25 µm in *S. perlongisporum* (Boidin and Gilles 1988). *S. tedersooi* also has shorter basidiospores and longer cystidia than its sister species *S. fusisporum* (see Figs 4, 10 and 11 for spore and cystidia comparisons and Figs 12–14 for phylogenetic inference).

### Sequence dissimilarities and barcoding gap

The aligned ITS dataset included 59 *Subulicystidium* sequences and two outgroup sequences of *Brevicellicium*. The dataset consisted of 671 characters (gaps included) and contained 477 distinct alignment patterns, namely 238 in ITS1, 28 in 5.8S and 211 in ITS2 region. The proportion of gaps and completely undetermined characters in this alignment was 20.02%.

**Figure 1.**
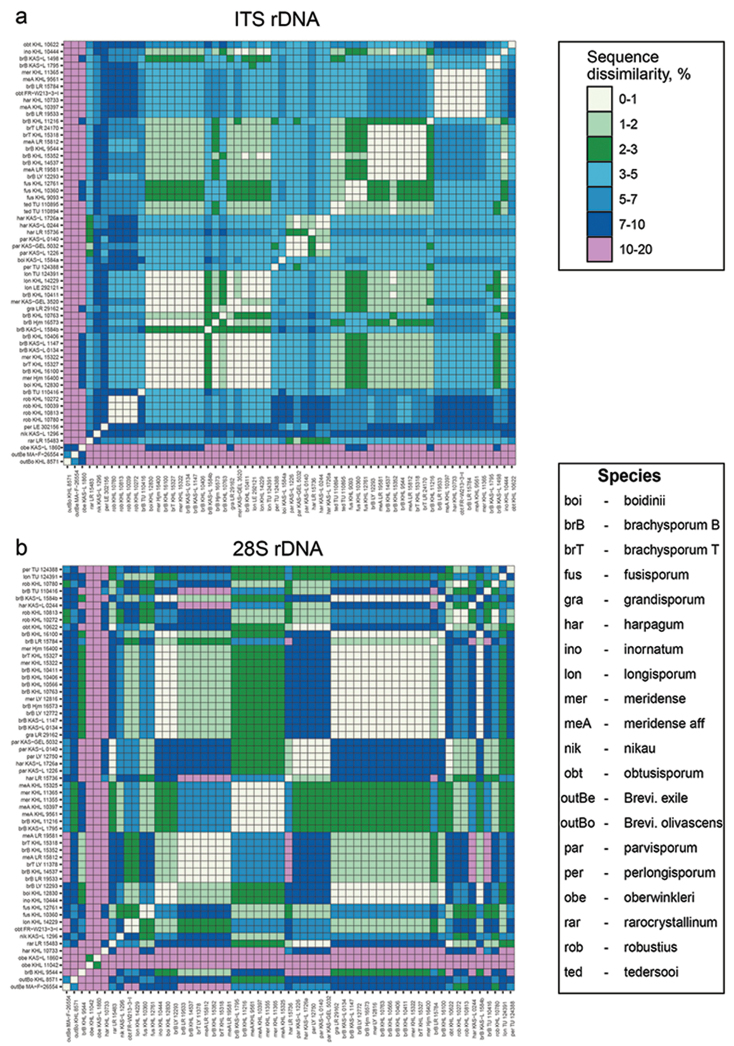
Raw pairwise dissimilarities (proportion of the differing sites, %) between *Subulicystidium* sequences of (**a**) ITS and (**b**) 28S region. Three-letter code before each specimen’s number corresponds to a species epithet as explained in the legend.

Aligned ITS sequences fell into several dissimilarity categories. All the *Subulicystidium* sequences were at least 10% different from two *Brevicellicium* sequences (outgroup), as well as from single sequence of *Subulicystidium oberwinkleri* (Fig. 1a). The sequences of *S. robustius* were at least 3% and maximum 10% different from the rest of the genus. The sequences of *S. harpagum* and *S. parvisporum* were most distant from *S. robustius* (7–10%) and 3-7% distant from the rest of the genus. Sequences mostly belonging to morphospecies *S. meridense* and *S. brachysporum* formed four groups within which they were all 0-3% (in many cases only up to 1%) dissimilar. One of these groups included also sequences of *S. fusisporum* and *S. tedersooi*, another group—*S. longisporum* and *S. grandisporum* and the third—single sequence of *S. obtusisporum*. Therefore, both easier and harder distinguishable species, in terms of ITS region identity, were found in the dataset.

**Figure 2.**
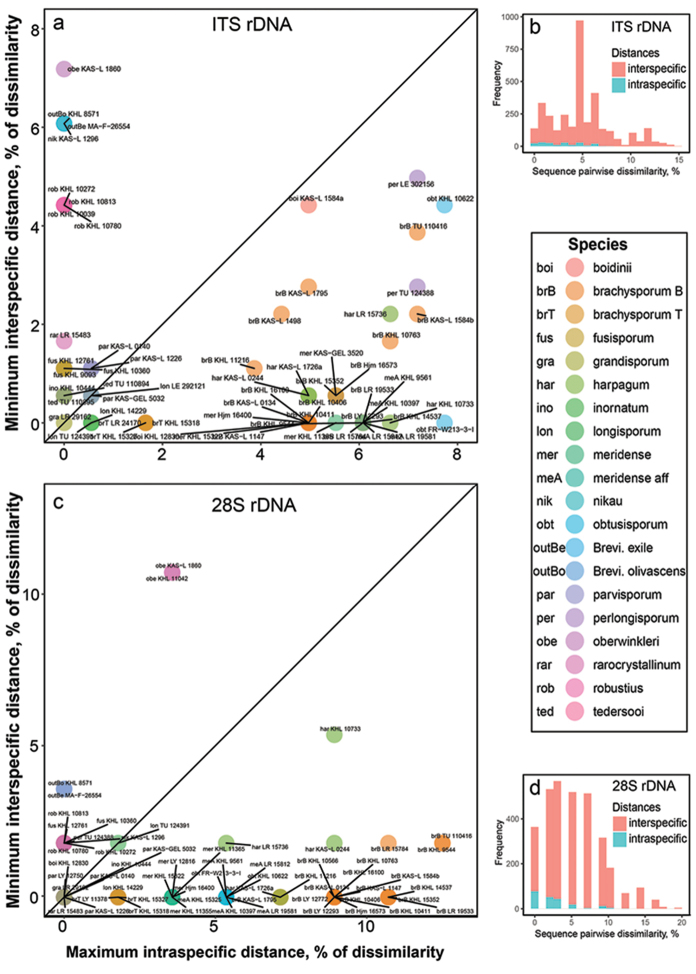
Verifying the presence of the barcode gap in *Subulicystidium* rDNA sequences of ITS (**a, b**) and 28S (**c, d**) regions. **a, c** Maximal intraspecific divergence compared with minimal interspecific distances between the aligned rDNA sequences in ITS (**a**) and 28S (**c**) datasets. Specimens falling above 1:1 line indicate the presence of the barcoding gap (molecular distinctness of the species) **b, d** Frequency distributions of intra- and interspecific distances without referring to particular species in ITS (**b**) and 28S (**d**) datasets. In the legend, the capital “B” following epithet in *S. brachysporum* means morphological species concept following Boidin and Gilles (1988), while “T” means the species as described by Talbot (1958). Three-letter code before each specimen’s number corresponds to a species epithet as explained in the legend

**Figure 4.**
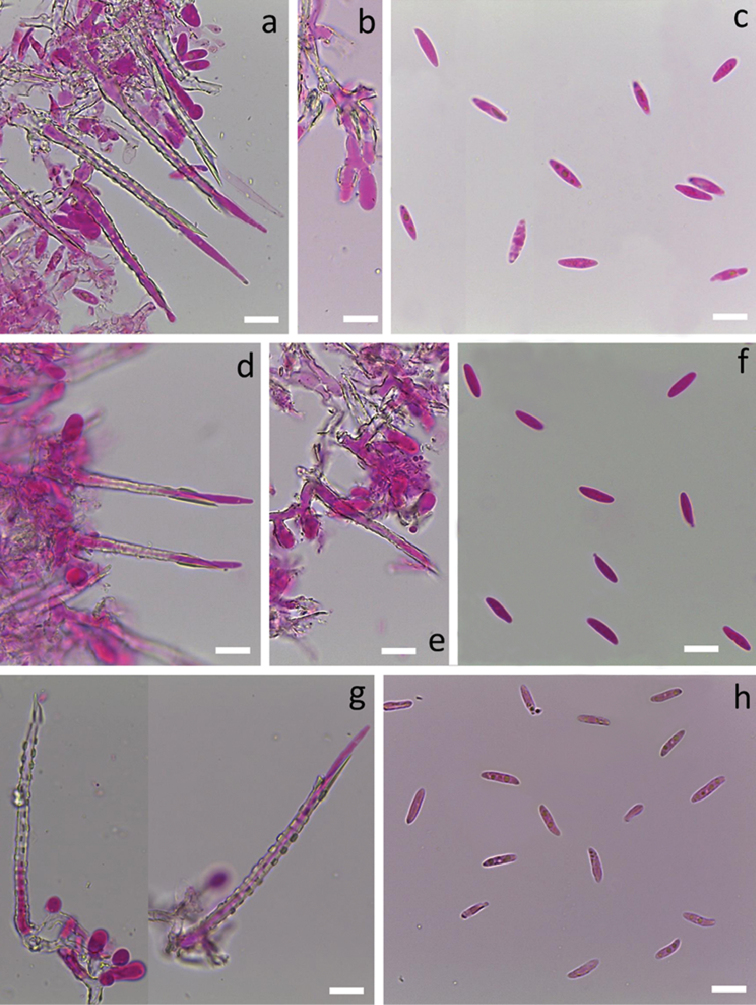
Species of *Subulicystidium* with narrow fusiform basidiospores. *Subulicystidium fusisporum* (GB:KHL 10360, holotype): **a** cystidia **b** crystalline encrustation of hymenium **c** basidiospores. *Subulicystidium inornatum* (GB:KHL 10444, holotype): **d** cystidia **e** young hymenium with slight overall encrustation **f** basidiospores. *Subulicystidium tedersooi* (TU 110894, holotype): **g** cystidia, h basidiospores. All preparations done in 3% aqueous solution of potassium hydroxide (KOH) mixed with 1% aqueous solution of Phloxine. All scale bars equal 10 µm.

The pattern seen through a visual inspection of the ITS sequence dissimilarity matrix was confirmed by the barcoding gap analysis. Throughout the dataset, intraspecific and interspecific distances strongly overlap and no universal for the genus *Subulicystidium* barcoding gap could be detected (Fig. 2b). Mean and maximal intraspecific distances were 2.87 and 7.73%, while mean and minimal interspecific distances were 5.06 and 0%, respectively. At the level of individual species, a barcode gap existed for *S. fusisporum*, *S. parvisporum*, *S. robustius* and *S. tedersooi* (Fig. 2a).

The aligned 28S dataset included 57 *Subulicystidium* sequences and two outgroup sequences of *Brevicellicium*. The dataset consisted of 617 characters (gaps included) and contained 246 alignment patterns, while the proportion of gaps and completely undetermined characters was 7.54%.

Pairwise 28S sequence dissimilarities were structured differently compared to the ITS dataset (Fig. 1b). The most distinct species in terms of 28S identity was *S. oberwinkleri.* The dissimilarity of its two sequences from the rest of *Subulicystidium* and two *Brevicellicium* sequences was 10–20%. The next most distinct group was formed by the sequences of *S. harpagum* and *S. parvisporum* which were 7–10% dissimilar from the rest of the genus except one group containing *S. meridense* and *S. brachysporum* sequences (2–3%). The majority of dissimilarities lay in the range 1–5% and were clearly grouped (Fig. 1b).

In a whole 28S dataset, intraspecific and interspecific distances strongly overlapped and thus showed no universal for the genus *Subulicystidium* barcode gap (Fig. 2d). Mean and maximal intraspecific distances were 2.52 and 12.5%, while mean and minimal interspecific distances were 5.58 and 0%, respectively. At the level of individual species, the barcode gap was evident for *S. oberwinkleri*, *S. fusisporum*, and *S. robustius* (Fig. 2c).

### Phylogenetic analyses

Bayesian analysis (BA) of the ITS alignment was finished with the standard deviation of split frequencies of 0.008 (equals average) and was characterised by the average potential scale reduction factor 1.00 (maximal 1.002) and pooled effective sample size from two MCMC runs 4151.3494. Maximum likelihood analysis (ML) resulted in a tree with a final optimisation log likelihood of -7019.372. BA produced a tree with a partly similar topology to ML tree but contained large polytomy at one of the basal nodes. Hereinafter we present and discuss the topology of the BA tree plotted with both posterior probabilities (pp) from BA and bootstrap supports (bs) from ML.

The phylogenetic tree, generated for the ITS dataset, contains monophyletic and polyphyletic taxa as well as several species represented by a single sequence (Fig. 12). *Subulicystidium oberwinkleri* is the most basal member of the ingroup. Other singletons descending from the basal nodes are *S. nikau*, the most deviating sequence of the morphospecies *S. brachysporum* (TU 110416) (see Discussion for the explanation) and *S. rarocrystallinum*. The clade dominated by Reunionese collections (pp=1, bs=87%) contains the new species *S. parvisporum* (pp=1, bs=99%) and *S. harpagum* (pp=1, bs=98%). The latter includes also L. Ryvarden’s collection from Colombia (LR 15736 in O:F: 918487). Two sequences of *S. perlongisporum* are also placed in the basal part of the tree but do not form a separate clade and, moreover, one sequence (from LE 302156) forms a clade with *S. boidinii* (KAS:L 1584a). *S. robustius* is recovered as a distinct clade (pp=1, bs=100%) of four sequences from Neotropics subtended by a long branch. *S. fusisporum*, represented by three sequences from the Caribbean region (pp=1, bs=98%), is a sister species to *S. tedersooi* represented by two sequences from Vietnam (branch support pp=1, bs=98%).

The remaining three clades each contain a mixture of sequences belonging to the morphospecies *S. brachysporum* and *S. meridense* with their likes. One clade contains also single sequences of *S. obtusisporum* from Germany (FR: W213-3-I) and *S. harpagum* from Jamaica (GB:KHL 10733). This clade is joined by three sequences of *S. brachysporum*: first by two sequences from Réunion (KAS:L 1498 and 1795) and at the next ancestor node with one sequence from Costa Rica (GB:KHL 11216). Another large clade is roughly equally rich in sequences of *S. brachysporum* and *S. meridense* (pp=1, bs=100%) and joined by a single sequence of *S. inornatum* (pp=0.88, bs=41%). One large clade (pp=1, bs=99%) included more collections of *S. brachysporum*, mostly sensu Boidin and Gilles (1988) and less of *S. meridense*, but also a single sequence of *S. grandisporum* (LR 29162 in O:F 506781). One more sequence of *S. brachysporum* (GB:KHL 10411) formed a weakly supported clade with the three sequences of *S. longisporum* from Europe (pp=0.84, bs<50%).

Bayesian analysis (BA) of the 28S alignment was finished with the standard deviation of split frequencies of 0.004 (equals average) and was characterised by the average potential scale reduction factor 1.00 (maximal 1.005) and pooled effective sample size from two MCMC runs 4673.55. Maximum likelihood analysis (ML) resulted in a tree with a final optimisation log likelihood of -2209.83. BA produced the tree with the topology highly similar to that of the ML tree. Hereinafter we present and discuss the topology of the BA tree plotted with both posterior probabilities (pp) from BA and bootstrap supports (bs) from ML, mostly focusing on differences from the results obtained for the ITS dataset.

The most basal ingroup members on the 28S tree were *S. oberwinkleri* (clade with two sequences, pp=1, bs=100%), *S. rarocrystallinum* and *S. harpagum* from Jamaica (GB:KHL 10733) (Fig. 13). The species *S. harpagum* and *S. parvisporum* remained in the single clade (pp=1, bs=92%) but were recovered as polyphyletic due to the placement of sequence from the KAS:L 1226 amongst the sequences of *S. harpagum*. They were joined by the clade containing sequences from *S. obtusisporum* (GB: KHL 10622) and *S. brachysporum* (TU 110416). The remaining sequences, mostly belonging to *S. brachysporum* and *S. meridense*, occupy upper nodes of the tree without clear grouping by morphospecies. 28S dataset was importantly enriched by the sequences from specimens, for which the ITS region could not be sequenced: GB:KHL 10566, O:F 506782 and LY 12772 (*S. brachysporum* sensu Boidin and Gilles), LY 11378 (*S. brachysporum* sensu Talbot), GB:KHL 11355 and LY 12816 (*S. meridense*), KHL 15325 (S. aff. meridense) and LY 12750 (*S. parvisporum*).

Bayesian and Maximum likelihood phylogenetic analyses of the concatenated alignment (ITS+28S) resulted in a tree topology which was contributed by both ITS and 28S regions (Fig. 14). In line with the ITS-based tree, *S. boidinii* and *S. harpagum* were recovered as polyphyletic but *S. parvisporum* as monophyletic (cf. Fig. 12). Furthermore, the German sequence of *S. obtusisporum* was found in the brachysporum-meridense clade and not between the sequences of *S. longisporum* (cf. Fig. 13). On the other hand, similarly to 28S-based tree, *S. fusisporum* and *S. tedersooi* intruded the brachysporum-meridense clades. Additionally, the 28S portion of concatenated alignment contributed to resolving a polytomy at one of the basal node containing sequences of *S. nikau* and *S. rarocrystallinum*.

### Spore-based species comparisons

We measured in total 2840 basidiospores from 67 specimens of *Subulicystidium*. We defined three groups of species according to the principal basidiospore shape: species with fusiform, cylindric and allantoid basidiospores. We found that some of the species could be delimited based on the basidiospore morphology solely, while, for other species, this was not possible and additional morphological characters had to be considered.

The species with fusiform basidiospores are barely distinguishable according to the basidiospore length. It varied generally from 8 to 11 µm, while the mean value did not exceed 10 µm (Fig. 11a). The only exception was *S. fusisporum* which had spores 10.7–12.3 µm long (main range, i.e. 5–95% quantiles of measurements data) and 11.5 µm long in average. Three species, viz. *S. robustius*, *S. inornatum* and *S. fusisporum* were indistinguishable in the spore width which varied for three of them between 2.5 and 3.5 µm. In contrast, *S. ryvardenii* had broader basidiospores with the main range 3.5–4.2 µm and mean value 3.8 µm. *S. naviculatum* was distinguished by the broadest fusiform basidiospores (main range 4.3–5.0 µm, mean value 4.6 µm), while *S. tedersooi* by the narrowest fusiform basidiospores (main range 2.1–2.6 µm, mean value 2.4 µm). The spore length to width ratio was as useful as spore width to discriminate the species and was remarkably the lowest in *S. naviculatum* (2.0–2.5, mean 2.2) and the highest in *S. tedersooi* (3.5–5.0, mean 4.2).

Under allantoid basidiospores, we considered those with adaxial side clearly concave and having length to width ratio around 2, thus looking rather as reniform or phaseoliform. Amongst the species with such spores, *S. oberwinkleri* had distinctly the longest and the broadest spores: mean length and width were 9.2 and 4.7 µm, respectively (Fig. 11b). *S. nikau* could be distinguished from *S. boidinii* by broader spores. The spore width in *S. nikau* was 3.9–4.6 µm (mean 4.2 µm) and length-width ratio is 1.6–2.0 (mean=1.8), while *S. boidinii* has spores 2.8–3.3 µm broad (mean 3.1 µm) and length-width ratio 1.9–2.4 (mean=2.1). Comparing our own data with the Cunningham’s specimen of *S. nikau* (holotype) and Boidin’s specimen of *S. boidinii* showed that we have the same understanding of the respective species as the mentioned authors. *S. harpagum* and *S. parvisporum* had rather overlapping spore width and length to width ratio but differed in the spore length: 5.6–8.3 µm (mean 6.7 µm) in the former versus 5.0–6.2 µm (mean 5.6 µm) in the latter.

Species with cylindric basidiospores (Fig. 11c) were characterised by the average length to width ratio of at least 2.8 and were well distinguished by the mean spore length: 12.7 µm in *S. grandisporum*, 10.7 µm in *S. obtusisporum* and 9.2 µm in *S. cylindrosporum*. The collections of the meridense-brachysporum morphogroup were also characterised by cylindrical basidiospores but of the smaller length and were less clearly distinguishable. Examples of single collections representing each morphogroup (Fig. 11d) showed that *S. brachysporum* sensu Boidin and Gilles (1988) had on average slightly longer basidiospores than *S. brachysporum* sensu Talbot (1958), viz. 7.9 µm versus 7.3 µm. Curved spores of the classical *S. meridense* were on average shorter than straight spores of S. aff. meridense, viz. 6.9 µm versus 8.2 µm. Spores in the type specimen of *S. meridense* were of the intermediate average length compared to the two former examples (7.4 µm). The spore width and length to width ratio were very much overlapping in the material of meridense-brachysporum morphogroup.

With reference to the newly obtained data, in the next section we present the key to the genus *Subulicystidium*. We used the successful key of Gorjon et al. (2011) as the basis and a source of the information on the long-spored taxa.

### Morphological key to the genus *Subulicystidium*

**Table:** 

1	Basidiospores acicular, Q>4.5	**2**
–	Basidiospores cylindrical, fusiform, allantoid to reniform, Q<4.5	**5**
2	Basidiospores 12–16 × 2–3 µm, Q=4.5–7	***longisporum***
–	Basidiospores longer, Q>7	**3**
3	Basidiospores spirally curved, 27–35 µm long	***curvisporum***
–	Basidiospores straight or only slightly curved, shorter	**4**
4	Basidiospores 20–27 × 2–3 µm, cystidial crystalline sheath ends with a bundle of needle-like crystals	***cochleum***
–	Basidiospores 16–25 × 1.5–2.5 µm, cystidia with regular ornamentation (rows of rectangular crystals)	***perlongisporum***
5	Basidiospores fusiform	**6**
–	Basidiospores cylindric to broad cylindric (straight or curved)	**11**
6	Basidiospores 4–5 µm wide	***naviculatum***
–	Basidiospores narrower	**7**
7	Cystidia almost smooth, without regular rectangular crystalline protrusions	***inornatum***
–	Cystidia with regular ornamentation (rows of rectangular crystals)	**8**
8	Cystidia with large crystalline protrusions	**9**
–	Cystidia with small to moderately large crystalline protrusions	**10**
9	Basidiospores 2.5–3.5 µm wide	***robustius***
–	Basidiospores broader than 3.5 µm	***ryvardenii***
10	Basidiospores 8.5–11.5 × 2–2.5 µm wide	***tedersooi***
–	Basidiospores 10.5–12.5 × 2.5–3.5 µm wide	***fusisporum***
11	Basidiospores broad cylindric, Q=1.5–2.5	**12**
–	Basidiospores cylindric, Q=2.5–4.5	**14**
12	Cystidia covered with irregularly shaped large crystalline plates, 80–150 × 5.5–10 µm, basidiospores 8–11 × 4.0–5.5 µm	***oberwinkleri***
–	Cystidia with regular ornamentation (rows of rectangular crystals) and smaller, basidiospores also smaller	**13**
13	Basidiospores 7–9 × 3.5–4.5 µm	***nikau***
–	Basidiospores 6–8 × 2.8–3.5 µm	***boidinii***
14	Basidiospores 3–4 µm wide	**15**
–	Basidiospores 2–3 µm wide	**17**
15	Basidiospores 10–15 µm long	***grandisporum***
–	Basidiospores shorter	**16**
16	Basidiospores 9–13 µm long, cystidia with regular rows of rectangular crystals	***obtusisporum***
–	Basidiospores 8–10.5 µm long, cystidia bear rectangular to rounded, rather sparse and irregularly arranged crystals	***rarocrystallinum***
17	Basidiospores 5.0–6.2 µm long	***parvisporum***
–	Basidiospores longer	**18**
18	Basidiospores 7–10.5 µm long	***brachysporum* sensu Boidin and Gilles (1988)**
–	Basidiospores 6–8 µm long	**19**
19	Crystal protrusions on cystidia are short rods that project backwards under acute angle, giving cystidia the resemblance of a harpoon	***harpagum***
–	Cystidia with regular ornamentation (rows of rectangular crystals)	**20**
20	Basidiospores elliptic with attenuated base, usually straight	***brachysporum* sensu Talbot (1958)**
–	Basidiospores cylindric, straight to regularly curved	***meridense***


## Discussion

### General remarks

In this study, we describe 11 new species of *Subulicystidium* based on morphological evidence and rDNA ITS and 28S sequence analyses. Ten of these species are characterised by a unique combination of basidiospore and cystidium morphology and rDNA sequence identity. One species (*S. ryvardenii*) could not be sequenced but the morphological evidence itself was sufficient for describing it as a new species. With our contribution, the number of the known species in the genus *Subulicystidium* now totals 20. The provided morphological key to all known species should facilitate identification of specimens previously treated as highly variable *S. longisporum* or left without species name. Such literature is urgently needed to assist in tropical fungal inventories.

We revised also the morphological and genetic borders of the five previously known species. One of them, *S. naviculatum*, could not be sequenced, while for *S. nikau*, only one specimen with amplifiable DNA was available. For the two morphospecies, *S. brachysporum* and *S. meridense*, numerous sequences from different localities were obtained. Our data show that, despite differences in the protologues, the species are hard to separate morphologically and molecularly. They share, to a large extent, basidiospore size and shape as well as highly similar ITS and 28S sequences, leading to strongly intermixed clades in phylogenetic trees. Therefore, *S. brachysporum* and *S. meridense*, in current understanding, are highly polyphyletic.

### Species distributions

Our study is based on examination of a large set of specimens from numerous localities in Paleo- and Neotropics. Upon this, we could show that the diversity of the short-spored *Subulicystidium* species is much higher than previously known. The newly described *S. robustius* is in fact a frequently occurring species in the Caribbean region and in South America. Furthermore, we could report a multicontinental distribution for several species, verified by DNA sequence data. *S. brachysporum*, *S. boidinii*, *S. harpagum* and *S. oberwinkleri* are typified by material from Paleotropics (South Africa in the first species and Réunion in three others), but were found by us also in South America. The morphospecies *S. meridense*, described from Venezuela (Oberwinkler 1977) and later found in Costa Rica (Kisimova-Horovitz et al. 1997), was also found on Réunion Island by Boidin and Gilles (1988). In addition to sequenced collections from a few more countries in South America, we confirmed the species presence on Réunion by sequencing collections of Boidin and Gilles (1988). In this study, we also report *S. meridense* for the first time from South-East Asia (Taiwan). The new species *S. fusisporum* was first considered by us as a Caribbean endemic. Re-identification as *S. fusisporum* of the specimen collected by G. Gilles in Côte D’ivoire (LY 7375, originally labelled as *S. longisporum*, DNA could not be amplified) may suggest that the species is present in West Africa as well.

It was surprising for us to find the species occurring on more than one continent or on islands separated by thousands of kilometres. For fungi with spores carried by wind, dispersal limitation was shown to act strongly even at small spatial scales (Peay et al. 2010, Norros et al. 2012). Given the architecture and location of *Subulicystidium* fruit-bodies (next to the ground, not rarely underside of the logs), one would expect prevailing spore dispersal distance smaller than 1 m (Galante et al. 2011). However, in macrofungi, there remains a probability of spore travel on a distance of kilometres (Nordén and Larsson 2000, Peay et al. 2012, Norros et al. 2014) and also overseas (Geml et al. 2012). Spore morphology traits have been recently discussed in a connection with dispersal and arrival success of a species (Norros et al. 2014, Calhim et al. 2018). In this regard, the genus *Subulicystidium*, with a high diversity of spore size and shape between species, is an interesting object to correlate spore traits and biogeography in future studies.

### Remarks on the previously known species

***Subulicystidium brachysporum* (P.H.B. Talbot & V.C. Green) Jülich**

Photos of fresh collections in PlutoF: link 1, link 2

Figs 8; 10l, m

**Figure 10.**
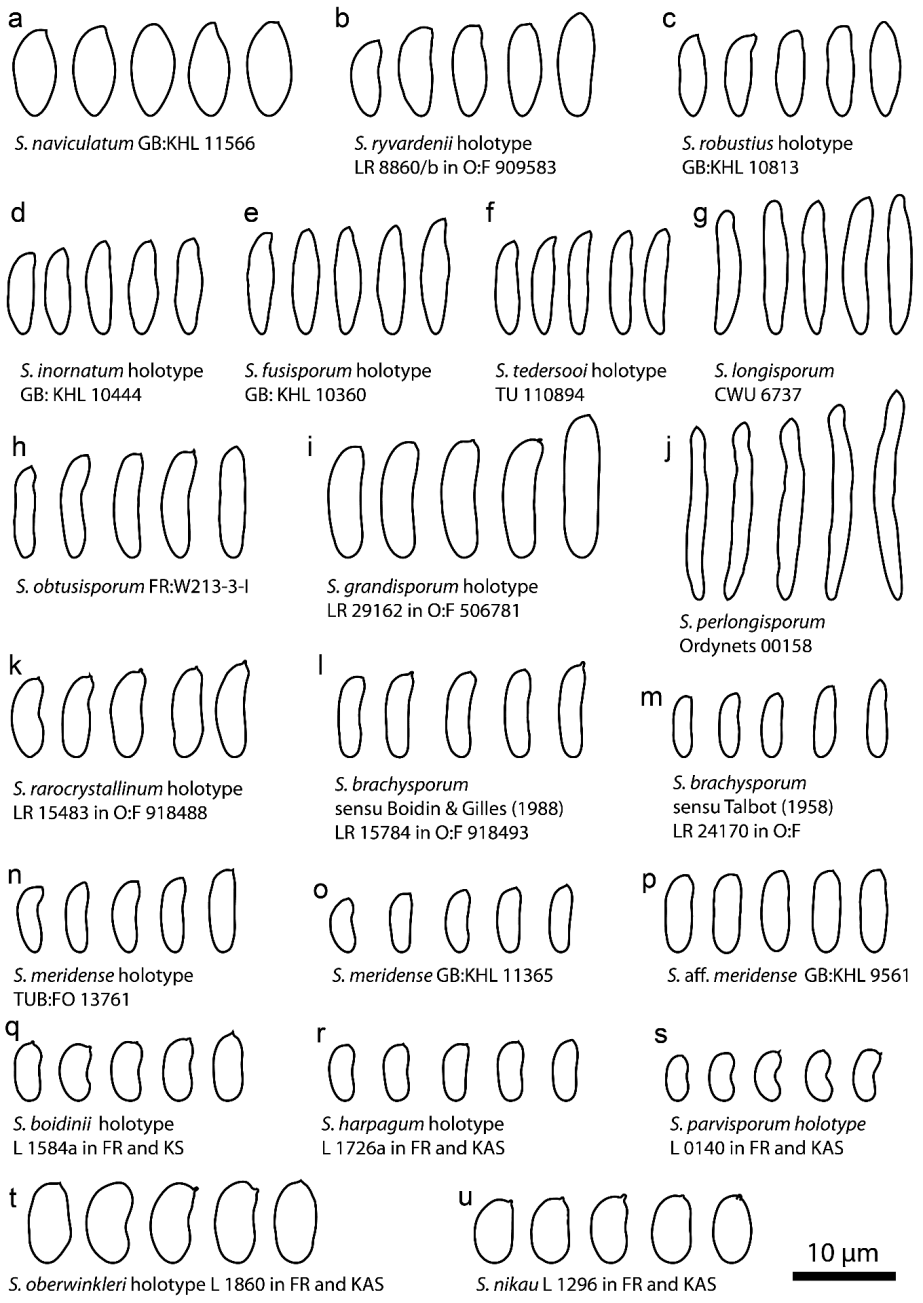
Basidiospore shape and size in all studied species of *Subulicystidium*. Each species is illustrated by a single specimen and herbarium codes are indicated on the figure.

**Figure 11.**
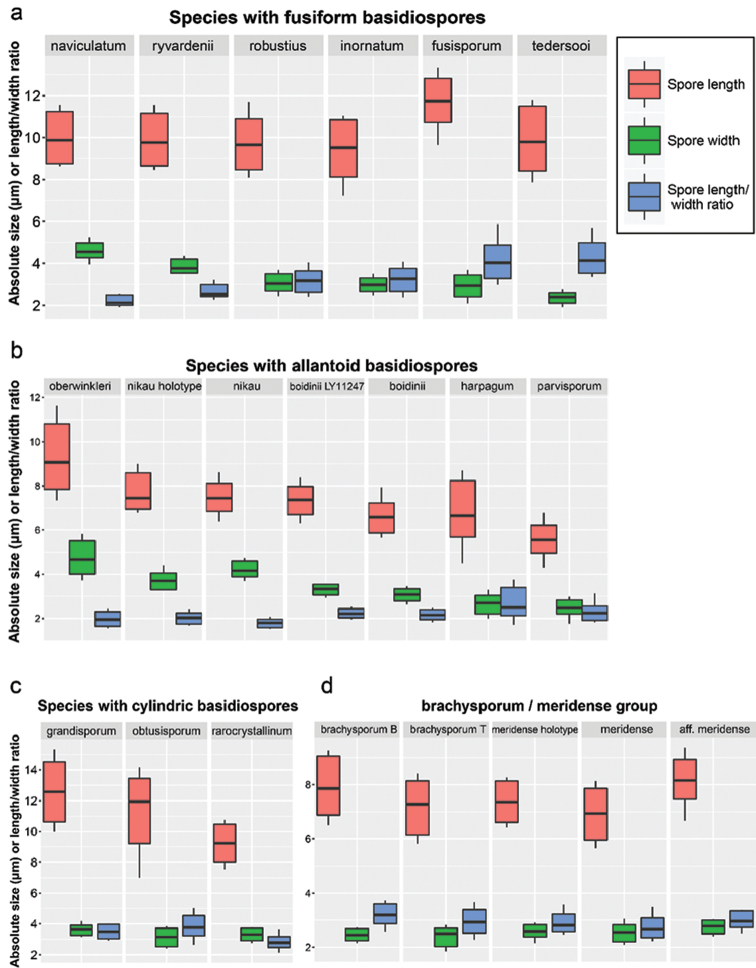
Basidiospore size range in the short-spored species of *Subulicystidium*. Only measurements from sequenced or important historical collections were included in calculations (in total 67 specimens, 2840 basidiospores). Boxes (with median inside) delimit the range between 5% and 95% data quantiles, while the whiskers show minimum and maximum values without considering outliers (see Materials and Methods for details on excluding outliers). If more than one sequenced specimen was available for species, raw measurements without outliers were pooled to calculate basidiospore size range of the species. In *S. brachysporum*, the capital “B” following epithet means morphological species concept following Boidin and Gilles (1988), while “T” means the species as described by Talbot (1958).

**Figure 12.**
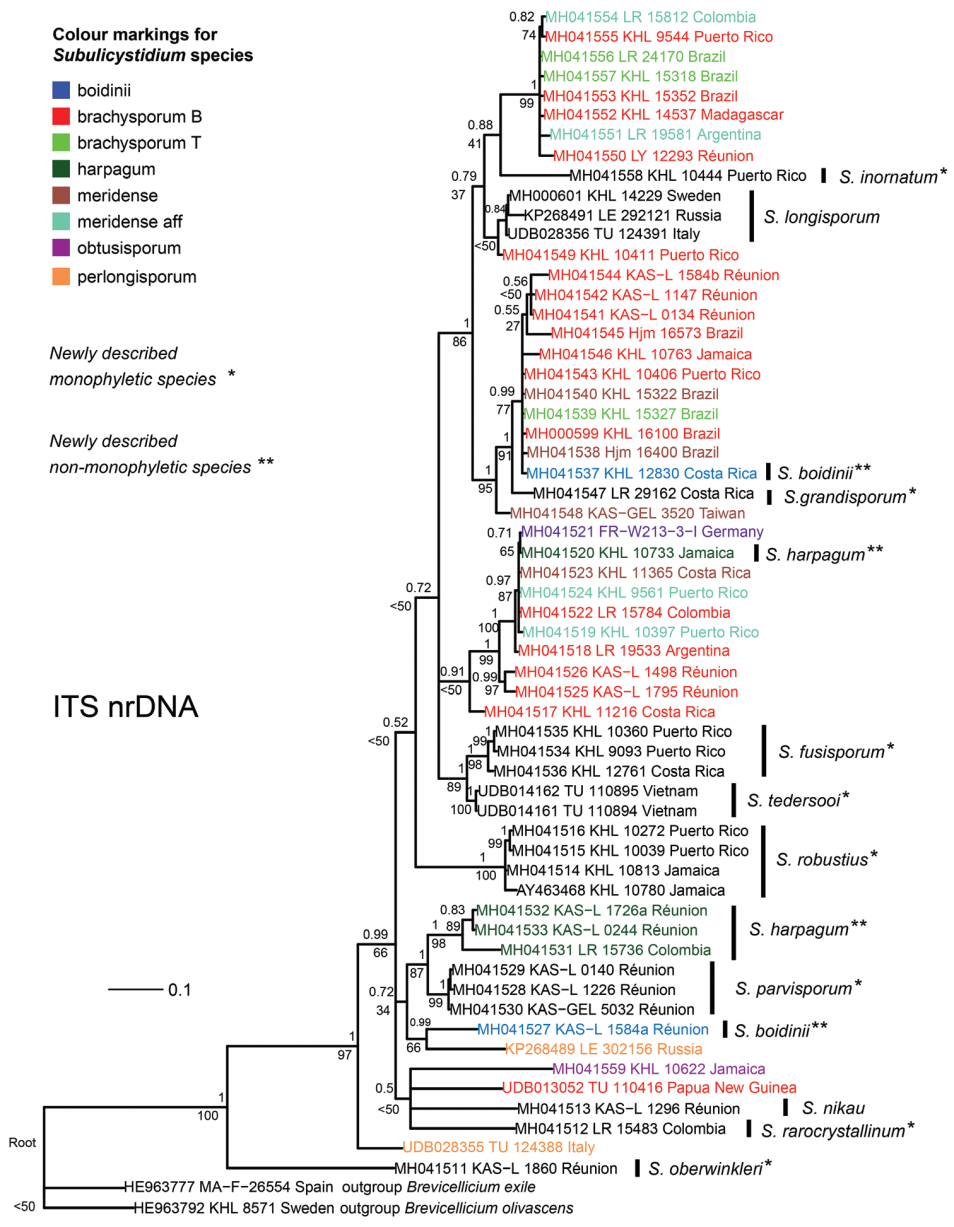
Phylogenetic relationship of *Subulicystidium* based on ITS nrDNA sequences. 50% majority-rule consensus tree from Bayesian analysis is shown, with posterior probabilities above the branches and bootstrap support values from the maximum likelihood estimation below the branches. Tips of the tree are annotated according to morphological identification and marked with colours in non-monophyletic taxa (see legend). In the legend, the capital “B” following epithet in *S. brachysporum* means morphological species concept following Boidin and Gilles (1988), while “T” means the species as described by Talbot (1958).

**Figure 13.**
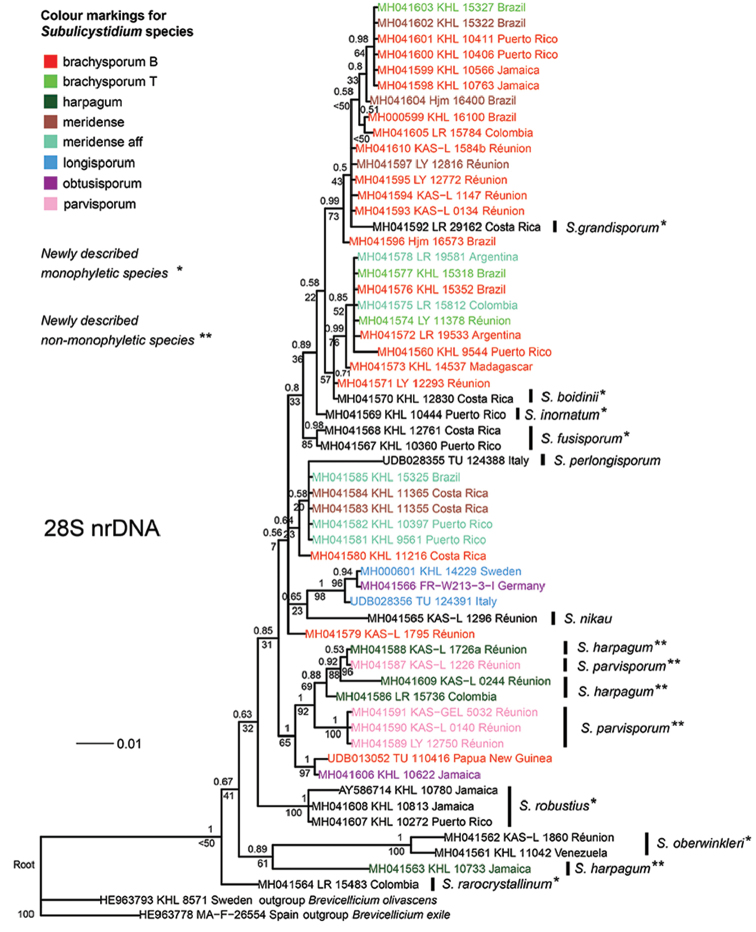
Phylogenetic relationship of *Subulicystidium* based on 28S nrDNA sequences. 50% majority-rule consensus tree from Bayesian analysis is shown, with posterior probabilities above the branches and bootstrap support values from the maximum likelihood estimation below the branches. Tips of the tree are annotated according to morphological identification and marked with colours in non-monophyletic taxa (see legend). In the legend, the capital “B” following epithet in *S. brachysporum* means morphological species concept following Boidin and Gilles (1988), while “T” means the species as described by Talbot (1958).

**Figure 14.**
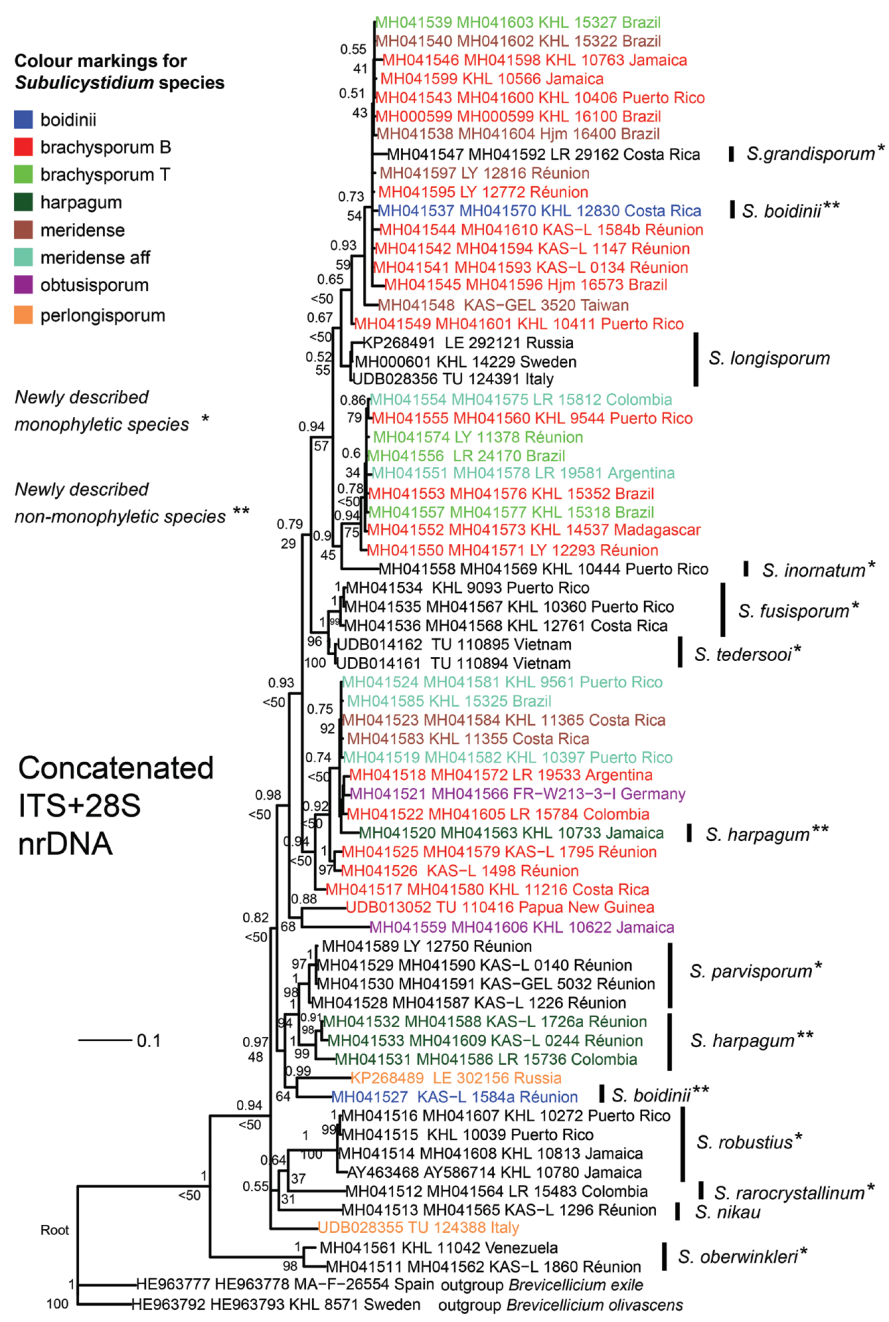
Phylogenetic relationship of *Subulicystidium* based on concatenated ITS+28S nrDNA alignment. 50% majority-rule consensus tree from Bayesian analysis is shown, with posterior probabilities above the branches and bootstrap support values from the maximum likelihood estimation below the branches. Tips of the tree include GenBank/UNITE accession numbers of ITS followed by 28S region. Tips are annotated according to morphological identification and marked with colours in non-monophyletic taxa (see legend). In the legend, the capital “B” following epithet in *S. brachysporum* means morphological species concept following Boidin and Gilles (1988), while “T” means the species as described by Talbot (1958).

**Notes.** Unfortunately, we were not able to study the type specimen of Peniophora longispora var. brachyspora (Talbot’s No. 40683 in PREM, Mycology Division, ARC-Plant Protection Research Institute, Queensland, South Africa). However, reviewing taxonomic literature on *Subulicystidium* brought us to delineating two morphogroups in the species *S. brachysporum*, according to the views of the earlier authors. Talbot (1958), while describing Peniophora longispora var. brachyspora, characterised its basidiospores as “elliptic-fusoid, 6.4–8 × 2.2–3.2 µm … sometimes with a faint band about the middle”. Boidin and Gilles (1988) described the basidiospores of *S. brachysporum* from Réunion as elliptic in frontal face and bananiform (cylindric with slightly attenuated apex, slightly curved) in lateral face, 7.5–10 × 2–2.5(–3) µm. Therefore, we differentiated groups of (i) *S. brachysporum* sensu Talbot, i.e. sensu typi, with straight oblong-elliptic basidiospores having long attenuated base, with the mean length below 7.5 µm and mean length to width ratio hardly reaching 3 (see Fig. 10m); and (ii) *S. brachysporum* sensu Boidin and Gilles with cylindric and slightly curved basidiospores with the mean length over 7.5 µm and length to width ratio between 3 and 4 (Fig. 10l). In our dataset, *S. brachysporum* sensu Boidin and Gilles was represented by far more specimens than *S. brachysporum* sensu Talbot.

The description and illustration of *S. brachysporum* by Duhem and Michel (2001) (specimen Bourdot No. 7986) and by Oberwinkler (1977, fig. 25, “authentical material”) correspond to the protologue and drawing of *S. brachysporum* by Talbot (1958). On the other hand, our examination of Venezuelan collection (FO15970 in TUB, see Oberwinkler 1977, fig. 29) let us assign it to *S. brachysporum* sensu Boidin and Gilles (1988). Descriptions by Liberta (1980), Maekawa (1998), Martini (2016) and illustrations by Eriksson et al. (1984, fig. 764 f, g – for single specimens from Canada and USA), match *S. brachysporum* sensu Boidin and Gilles (1988) as well.

DNA sequence similarity analyses and phylogenetic reconstructions did not support the presence of two morphogroups of *S. brachysporum*. Sequences of both morphotypes occured in the same clade and, moreover, shared the clade with sequences from the morphospecies *S. meridense*. For the moment, we prefer to retain two morphogroups of *S. brachysporum*, thus promoting further exploration of species limits and examination of the type specimen.

The most comprehensive overview of global occurrence of *S. brachysporum* was provided by Martini (2016). We can add that the species is present also in South America, Caribbean region and Madagascar.

**Specimens examined: *Subulicystidium brachysporum* sensu Talbot (1958).** ARGENTINA. Misiones: Iguazu National Park, Cataratas de Iguazu, -25.6748, -54.4532, on dead wood of angiosperm tree, 1-5 Mar 1982, L.Ryvarden (LR 19687 in O:F 506779). BRAZIL. Rondonia: Porto Velho, Rua Rio Madeira 7014, Nova Esperanca, -8.7160, -63.8785, on dead wood of angiosperm tree, 11 Mar 2012, K.-H.Larsson (KHL 15318 and 15327 in O:F). Sao Paulo: Santos, Ubatuba, Ilha Anchieta, -23.5500, -45.0667, on dead wood, 17-18 Jan 1987, D.Pegler, K.Hjortstam & L.Ryvarden (LR 24170 and LR 24203 in O:F). COLOMBIA. Magdalena: Parque Nacional Tayrona, Estacion de Gairaca, 0-30 m, 11.3170, -74.1063, on dead wood, 12 Jun 1978, L.Ryvarden (LR 15755 in O:F 918492). RÉUNION. Saint-Paul: Saint-Paul, St-Gilles-II, Ravine de St. Gilles, abandoned orchard, 200 m, on dead wood, 26 Apr 1985, J.Boidin (LY 11378).

**Specimens examined: *Subulicystidium brachysporum* sensu Boidin and Gilles (1988).** ARGENTINA. Misiones: Iguazu National Park, Cataratas de Iguazu, -25.6748, -54.4532, on dead wood of angiosperm tree, 1-5 Mar 1982, L.Ryvarden (LR 19533 in O:F 506782). BRAZIL. Pará: Belem, Museo Goeldii Scientific Centre, -1.4525, -48.4764, on dead wood, 25 Nov 2013, K.-H.Larsson (KHL 16461 in O:F). Paraiba: Areia, Reserva Estadual Mata do Pau-Fero, -6.9642, -35.7496, on dead wood of angiosperm tree, 28 Apr 2013, K.-H.Larsson (KHL 16100 in O:F). Rondonia: Porto Velho, Rua Rio Madeira 7014, Nova Esperanca, -8.7160, -63.8785, on dead wood of angiosperm tree, 11 Mar 2012, K.-H.Larsson (KHL 15330 in O:F); Rubber plantation park, -8.7324, -63.9008, on dead wood of angiosperm tree, 11 Mar 2012, K.-H.Larsson (KHL 15352 in O:F). Sao Paulo: Sao Paulo, Instituto de Botanica, -23.6450, -46.6261, on dead wood, 24 Jan 1987, K.Hjortstam (Hjm 16573 in GB). Colombia. Magdalena: Parque Nacional Tayrona, Estacion de Gairaca, 0-30 m, 11.3170, -74.1063, on dead wood, 12 Jun 1978, L.Ryvarden (LR 15784 in O:F 918490; LR 15744B in O:F 918491; LR 15784 in O:F 918493). COSTA RICA. Alajuela: Bijagua, Albergue Heliconias, Sendero Heliconias, 770 m a.s.l, 10.7181, -85.0453, on branch of angiosperm tree, 12 Jul 2001, K.-H.Larsson (KHL 11216 in GB); La Fortuna de San Carlos, Parque Nacional Volcán Arenal, Sendero Pilón, 650 m, 10.4589, -84.7644, on dead wood of angiosperm tree, 15 Jul 2001, K.-H.Larsson (KHL 11419 in GB). Puntarenas: Coto Brus, Sabalito, Zona Protectora Las Tablas, La Neblina, 8.9149, -82.7719, on stem of angiosperm tree, 5 Nov 2004, K.-H.Larsson (KHL 12764 in GB). DOMINICAN REPUBLIC. Provincia La Altagracia: Parque Nacional del Este, Playa Guaraguao, 18.3269, -68.8092, on dead wood, 3 Jun 1997, K.-H.Larsson (KHL 9920 in GB). ETHIOPIA. Oromia: West Shewa Zone, Ginchu, Chilomo Forest, 9.0900, 38.1700, on dead wood, 10 Jul 1990, L.Ryvarden (LR 28047 in O:F 909592). JAMAICA. Cornwall County: Trelawny parish, Cockpit Country, Ramgoat Cave, 350 m a.s.l., 18.3378, -77.5568, on twigs of angiosperm tree, 11 Jun 1999, K.-H.Larsson (KHL 10686 in GB); N of Crowlands, trail/road into park area, 18.2611, -77.6511, on branch of angiosperm tree, 10 Jun 1999, K.-H.Larsson (KHL 10633 in GB); Windsor Cave, along trail to Troy, 18.3564, -77.6472, on branch of angiosperm tree, 13 Jun 1999, K.-H.Larsson (KHL 10763 in GB). Middlesex County: Manchester parish, Marshalls Pen, Sutton Farm, 18.0589, -77.5308, on branches of angiosperm tree, 8 Jun 1999, K.-H.Larsson (KHL 10505 in GB), on stem of angiosperm tree, 8 Jun 1999, K.-H.Larsson (KHL 10566 in GB). Surrey County: Portland parish, between reach and Ecclesdown hillside to the east, 500 m, 18.0433, -76.3108, on branch of angiosperm tree, 16 Jun 1999, K.-H.Larsson (KHL 10855 in GB). MADAGASCAR. Anosy: Tolagnaro, Mandena Conservation Zone, -24.9529, 47.0028, on dead wood of angiosperm trunk, 10 Mar 2010, K.-H.Larsson (KHL 14359 and 14365 in O:F); Nahampoana, -24.9667, 46.9667, on dead wood of angiosperm trunk, 12 Mar 2010, K.-H.Larsson (KHL 14390 in O:F); Petriky Conservation Zone, -25.0667, 46.8500, on dead wood of angiosperm trunk, 14 Mar 2010, K.-H.Larsson (KHL 14486, 14505 and 14516 in O:F); Sainte Luce Conservation Zone, -24.8028, 47.1636, on dead wood of angiosperm trunk, 15 Mar 2010, K.-H.Larsson (KHL 14537 in O:F). Ihorombe: Isalo National Park, Namaza, along track from entrance to waterfall, -22.5583, 45.4000, on dead wood of angiosperm trunk, 7 Mar 2010, K.-H.Larsson (KHL 14293 in O:F). PAPUA NEW GUINEA. Morobe: Bawituc, Lae 12-Mile, Uphill of Bawituc, -6.6390, 146.9089, on dead twig, 3 Jul 1905, L.Tedersoo (TU 110416). PUERTO RICO. Municipio Isabela, Moñtanas Aymamón, Bosque Estatal de Guajataca, Verada Nueva Trail, 230 m, 18.4242, -66.9678, on dead fruitbodies of polypore, 26 Jun 1996, K.-H.Larsson (KHL 9544 in GB). Municipio Luquillo, Luquillo Mts, Bisley Experimental Watersheds, along the logging road, 215 m, 18.3161, -65.7467, on log, 6 Jun 1998, K.-H.Larsson (KHL 10270 in GB); Sabana, above Chicken Farm & Rio Sabana, 70 m, 18.3500, -65.7344, 10 Jun 1998, on woody branch, K.-H.Larsson (KHL 10406 in GB) and on wet log, K.-H.Larsson (KHL 10411 in GB), on wood of angiosperm tree, 7 Jun 1997, K.-H.Larsson (KHL 10088, 10095 and 10097 in GB). RÉUNION. Saint-Benoît: La Plaine-des-Palmistes, Palmistes III-87, en descendant vers St Benoit, alt 800 m, on dead wood, 23 Mar 1987, G.Gilles (LY 12456); Salazie, Hell Bourg, ca 1000 m, -21.0642, 55.5269, on dead branch, 23 Mar 2015, M.Striegel (L 1584b in FR and KAS). Saint Pierre: Cilaos, Cirque de Cilaos, Roche Merveilleux, Sentiere botanique, 1300 m, -21.1232, 55.4920, on strongly decayed wood, 15 Mar 2013, E.Langer (L 0134 in FR and KAS); L’Étang-Salé, Étang-Salé-87: forêt domaniale, alt 60-80 m, on dead wood of Fabaceae tree, 14 Mar 1987, G.Gilles (LY 12293); Saint-Philippe, Baril-II-87, depart du sentier botanique, 100-150 m, on dead branch, 6 Apr 1987, G.Gilles (LY 12772), Forêt de Mare Longue, 495 m, -21.3438, 55.7410, on dead branch, 28 Mar 2015, M.Striegel (L 1795 in FR and KAS); Saint-Pierre, Piton de Mont Vert, ca 560 m, -21.3279, 55.5413, on dead wood, 18 Mar 2015, M.Striegel (L 1498 in FR and KAS), Piton de Mont Vert, hiking path, ca 560 m, -21.3279, 55.5413, on dead branch, 18 Mar 2015, J.Riebesehl & M.Schroth (L 1147 in FR and KAS). VENEZUELA. Estado Amazonas: Manapiare, Yutajé, 110 m, 5.6142, -66.1236, on dead wood of angiosperm tree, 12-19 Jun 1997, L.Ryvarden (LR 40650A in O:F 909582). Estado Merida: Merida, 8.6249, -71.1395, on dead wood, 1969, F.Oberwinkler (FO 15970 in TUB).

***Subulicystidium longisporum* (Pat.) Parmasto**

**Note.** The following specimen was used to illustrate the species on Fig. 10g:

UKRAINE. Zakarpatska: Carpathian Biosphere Reserve, vicinities of Mala Uholka village, 670 m, 48.2632, 23.6175, on decayed deciduous wood, 11 Sep 2013, A.Ordynets (CWU 6737).

***Subulicystidium meridense* Oberw.**

Figs 9; 10n–p

**Notes.** According to Oberwinkler (1977), the basidiospore size of the type collection (FO13761 in TUB) is 6–8 × 2.5–3 µm, suggesting the mean basidiospore length, width and their ratio are 6.5 µm, 2.75 µm and 2.36, respectively. Our re-measuring revealed longer basidiospores, namely 6.6–8.1 × 2.4–2.8 µm, with the length to width ratio 2.6–3.2 (2.9) (see Supplementary files 2-4).

When describing *S. meridense*, Oberwinkler (1977) stressed the importance of allantoid, i.e. clearly curved, basidiospores. We adhered to this concept assigning our collections to *S. meridense* (see Figs 10 n, o). We named those with similar spore size but with straight cylindric spores “Subulicystidium aff. meridense” (Fig. 10p). However, molecular level sequence similarity analyses and phylogenetic reconstructions did not support the presence of two distinct groups.

In addition to the South American and Reunionese specimens, we examined also specimens of *S. meridense* from India and Taiwan. Boidin and Gilles (1988) reported LY 12456 and LY 12772 from Réunion as *S. meridense* with the note that the spores are larger and more elongated than in the Venezuelan type material. Our morphological examination (and DNA sequence data for LY 12772) showed that the Reunionese collection represent *S. brachysporum* sensu Boidin and Gilles. However, we concur with Boidin and Gilles that LY 12816 (sequenced) from Réunion and LY 9144 from Gabon should be named *S. meridense*. The description of the specimen TMI 25520 from Vanuatu (Maekawa 2002) corresponds to our concept of Subulicystidium aff. meridense.

**Specimens examined: Subulicystidium aff. meridense**. ARGENTINA. Misiones: Iguazu National Park, Cataratas de Iguazu, -25.6748, -54.4532, on wood of angiosperm tree, 1-5 Mar 1982, L.Ryvarden (LR 19581 in O:F). BRAZIL. Rondonia: Porto Velho, Rubber plantation park, -8.7324, -63.9008, on dead wood of angiosperm tree, 11 Mar 2012, K.-H.Larsson (KHL 15325 in O:F). Sao Paulo: Santos, Ubatuba, Ilha Anchieta, -23.5500, -45.0667, on dead wood, 17-18 Jan 1987, D.Pegler, K.Hjortstam & L.Ryvarden (LR 24201 in O:F). COLOMBIA. Magdalena: Parque Nacional Tayrona, Estacion de Gairaca, 0-30 m, 11.3170, -74.1063, on dead wood, 12 Jun 1978, L.Ryvarden (LR 15812 in O:F 918846). PUERTO RICO: Municipio de Cerro Alto, Montanas Aymamon, limestone magote near Parador Guajataca, 60 m, 18.4828, -66.9583, on strongly decayed log of angiosperm tree, 27 Jun 1996, K.-H.Larsson (KHL 9561 in GB). Municipio Luquillo, Luquillo Mts, Sabana, above Chicken Farm & Rio Sabana, 70 m, 18.3500, -65.7344, on log, 10 Jun 1998, K.-H.Larsson (KHL 10397 in GB).

**Specimens examined: *Subulicystidium meridense*.** ARGENTINA. Misiones: Iguazu National Park, Cataratas de Iguazu, -25.6748, -54.4532, dead wood of angiosperm tree, 1-5 Mar 1982, L.Ryvarden (LR 19688 in O:F 506784). BRAZIL. Rondonia: Porto Velho, Rua Rio Madeira 7014, Nova Esperanca, -8.7160, -63.8785, on dead wood of angiosperm tree, 11 Mar 2012, K.-H.Larsson (KHL 15322 in O:F). Sao Paulo: Santos, Ubatuba, Ilha Anchieta, -23.5500, -45.0667, on decayed wood, 17-18 Jan 1987, D.Pegler, K.Hjortstam & L.Ryvarden (Hjm 16400 in GB). CENTRAL AFRICAN REPUBLIC. Lobaye: Nola, Boukoko, 3.8929, 17.9153, on dead tree trunk, 18 May 1965, J.Boidin (in LY 5476). COSTA RICA. Guanacaste: Area cons. Tempisque, Reserva Biologica Lomas Barbudal, near the entrance, 20-40 m, 10.5103, -85.3744, on dead wood of angiosperm tree, 14 Jul 2001, K.-H.Larsson (KHL 11355, 11365 and 11368 in GB). Puntarenas: Coto Brus, Sabalito, Zona Protectora Las Tablas, Progreso, Camino a Cotoncito, 1560 m, 8.9306, -82.8031, on wood of angiosperm tree, 3 Nov 2004, K.-H.Larsson (KHL 12557 in GB), La Neblina, 1350 m, 8.9149, -82.7719, on wood of angiosperm tree, 5 Nov 2004, K.-H.Larsson (KHL 12732 in GB). San José: Dota, San Gerardo, around Hotel Savegre, ca 2000 m, 9.5643, -83.8016, on branch of angiosperm tree, 9 Nov 2004, K.-H.Larsson (KHL 12969 in GB). GABON. Estuaire: Libreville, Bush littoral, km 13 N Libreville, 0.5338, 9.4673, on bark of dead wood, 2 Feb 1979, G.Gilles (LY 9144). INDIA. Darjeeling: Sukna, About 4 km from Sukna towards RongTong, 26.8246, 88.3625, on bark of dead/decaying branch of angiosperm, 9 Aug 1980, G.S.Dhingra (19201 in O:F 909586). RÉUNION. Saint-Benoit: Saint-Benoit, Route forestière 3 de Takamaka, -21.1038, 55.5724, on dead wood, 8 Apr 1987, G.Gilles (LY 12816). TAIWAN. Nantou: Huisun Recreation Area, path to the “stone frog”, 24.0912, 121.0337, on dead wood, 26 Apr 1996, G.Langer, E.Langer & C.-J.Chen (GEL 3520 and 3530 in KAS). VENEZUELA. Estado Aragua: Maracay, National Park Henri Pittier, Rancho Grande, 10.3800, -67.6190, on hardwood, 22 Jun 1995, L.Ryvarden (LR 35544/C in O:F). Estado Merida: Merida, Vicinities of Instituto Forestal Latino-Americano, 8.6249, -71.1395, on dead twigs, 27 Nov 1968, F.Oberwinkler (FO 13761 in TUB, holotype).

***Subulicystidium naviculatum* Oberw.**

Figs 3a, b; 10a

**Notes.** We examined a single collection from Costa Rica (KHL 11566 in GB) which had broad fusiform basidiospores (8.6–)8.8–11.2(–11.6) × (4.0–)4.3–5.0(–5.3) µm, i.e. slightly shorter than in the holotype specimen FO 12778 (TUB) from Venezuela: 10–12 × 4.5–5 µm (Oberwinkler 1977). Furthermore, the cystidia in our specimen were covered with rows of rectangular crystals while the ornamentation pattern of cystidia in the holotype resembled that of *S. harpagum*, i.e. short rod-like protrusions that project backwards under an acute angle, giving cystidia the resemblance of a harpoon (see fig. 32 in Oberwinkler 1977). Unfortunately, the holotype in TUB could not be located.

Kisimova-Horovitz et al. (1997) reported a collection from Costa Rica (207a-I, =FO 42968 in TUB) as *S. naviculatum.* However, we re-identified the collection as *S. robustius*.

**Specimens examined.** COSTA RICA. San José: Reserva Los Santos, Cerro de la Muerte, 1.5 km from Interamerican Highway along road to San Gerardo de Dota, 2850 m, 9.5964, -83.7986, on stem of angiosperm tree, 18 Jul 2001, K.-H.Larsson (KHL 11566 in GB).

***Subulicystidium nikau* (G. Cunn.) Jülich**

Figs 5c,d; 10u

**Notes.** The species was described by Cunningham (1955) as *Peniophora sororia* based on material from the midribs of the dead leaves of nikau palm (*Rhopalostylis sapida*) which is endemic to New Zealand. Later Cunningham (1963) noticed that the name was occupied (Bourdot and Galzin 1912; p. 386) and provided the legitimate name *Peniophora nikau*.

The holotype of *S. nikau* (PDD 13816) has basidiospores 7–9 × 4–5 µm as reported by (Cunningham 1955). We measured the holotype basidiospores as slightly narrower, viz. (6.8–)6.9–8.6(–9.0) × 3.3–4.1(–4.4) µm (see Supplementary files 2–4), confirming the measurements by Oberwinkler (1977) and Liberta (1980).

As the basidiospores are similar, *S. nikau* has been confused with *S. oberwinkleri*. The former has cystidia with regular ornamentation (rows of rectangular crystals) typical for the genus *Subulicystidium* and the generitype *S. longisporum.* In contrast, *S. oberwinkleri* has larger cystidia with large, irregularly shaped crystalline plates. Cunningham (1955) illustrated cystidia of *S. nikau* correctly, while the characterisation by Stalpers and Buchanan (1991) as “covered with plate-like crystals” is misleading. Oberwinkler (1977) and Maekawa (1998) realised the discrepancies in cystidial ornamentation but did not provide a solution. We re-identified the record of *S. nikau* from Reunion, LY12488 (Boidin and Gilles 1988) as *S. oberwinkleri*. We also collected and sequenced a Reunionese specimen with regularly ornamented cystidia (KAS: L1296). Though sampled on dead wood and far from locus classicus, we keep it under the name *S. nikau* for the time being. Réunion is thus the second known locality of the species after New Zealand. The record from Venezuela reported by Liberta (1980) has not been studied and is hard to interpret because no illustration of cystidia was provided.

**Specimens examined.** RÉUNION. Saint-Pierre: Saint-Philippe, Sentier de Takamaka, ca 840 m, -21.0913, 55.6199, on dead wood, 26 Mar 2015, J.Riebesehl & M.Schroth (L 1296 in FR and KAS). NEW ZEALAND. Auckland: Cascades, Waitakere Ranges, on dead leaf midribs of palm *Rhopalostylis sapida*, 3 Apr 1954, S.D.Baker (PDD 13816, holotype).

***Subulicystidium obtusisporum* Duhem & H. Michel**

Figs 6d, e; 10h

**Notes.** Duhem and Michel (2001) identified a collection from Venezuela (Oberwinkler 1977, fig. 29, FO15970 in TUB) as *S. obtusisporum*, while we regard the same specimen as *S. brachysporum* sensu Boidin and Gilles (1988). Maekawa (2002) reported *S. obtusisporum* from Vanuatu and Ghobad-Nejhad et al. (2009) from Russian Caucasus. Here we report the species from East Asia and Caribbean region.

The first sequenced material of *S. obtusisporum* is our collection from Frankfurt am Main, central Germany (FR:W213-3-I). Fruit-body morphology, as well as microhabitat (exposed dead wood) agree with the data for the type specimen and related collections from southern France (Duhem and Michel 2001). Another sequenced specimen GB:KHL 10622 from Jamaica was very distant from the German specimen in terms of ITS and 28S sequence identity and position on the phylogenetic tree, which means *S. obtusisporum* is polyphyletic. Sequencing additional specimens, not least from Asia, is needed to clarify the taxonomy of this morphospecies.

**Specimens examined.** CHINA. Jilin: Chang Bai Shan Forest Reserve, Hangcong hou, 750 m, on dead wood of *Acer sp.*, 11-17 Sept 1983, L.Ryvarden (LR 21774 in O:F 909590). COSTA RICA. Guanacaste: Area cons. Tempisque, Reserva Biologica Lomas Barbudal, near the entrance, 20-40 m, 10.5103, -85.3744, on deadwood of angiosperm tree, 14 Jul 2001, K.-H.Larsson (KHL 11373 in GB). GERMANY. Hesse: Frankfurt, Science Park at the Campus Riedberg of Frankfurt University, 50.1701, 8.6300, on decayed trunk, 31 Mar 2016, O.Koukol (W213-3-I in FR). ITALY. Italy. Latina: Circeo Natural park, Selva de Circeo, 41.3429, 13.0534, on wood of *Quercus sp.*, 22-25 Oct 1984, K.Hjortstam, K.-H.Larsson & L.Ryvarden (LR 22458 in O:F 505520). JAMAICA. Cornwall County: Trelawny parish, Crown Lands, trail/road into park area, 18.2611, -77.6511, on branches of angiosperm tree, 10 Jun 1999, K.-H.Larsson (KHL 10622 in GB). PUERTO RICO. Municipio Luquillo, Luquillo Mts, Bisley Experimental Watersheds, along track from parking place, 215 m, 18.3161, -65.7467, on branch of angiosperm tree, 6 Jun 1997, K.-H.Larsson (KHL 9955 in GB). TAIWAN. Chiayi: Shi Ding, road No. 18 in direction to Alishan at km 60, ca 1500 m alt, 23.4801, 120.4491, on dead wood, 1 May 1996, G.Langer, E.Langer & C.-J.Chen (GEL 3677 in KAS). Miaoli: Sheipa National Park, Kuanwu, forest ca 500 m in direction of Le Shan, trail on the left side of the road, ca 2100 m, 24.3624, 121.1252, on dead wood, 19 Apr 1996, G.Langer, E.Langer & C.-J.Chen (GEL 3409 in KAS).

***Subulicystidium perlongisporum* Boidin & Gilles**

**Notes.** The following specimen was used to illustrate the species on Fig. 10j: GERMANY. Hesse: Biedenkopf, little valley of Martinsbach creek, mixed forest 280 m, 50.5410, 8.3037, on dead wood, 23 Apr 2016, A.Ordynets & M.Theiss (Ordynets 00158 in KAS).

### Which morphological characters are useful for the species delimitation in *Subulicystidium*? 

In general, spore size and shape are of crucial importance for the taxonomy of fungi (Parmasto et al. 1987). In our study, however, we found that basidiospore morphology itself may be insufficient for species-rank identifications in *Subulicystidium*. In this regard, the usefulness of other morphological characters is worth discussing.

Jülich (1975) studied cystidia of *S. brachysporum* and *S. longisporum* under a scanning electron microscope and concluded the identity of their ornamentation pattern. Jülich (1975) hypothesised, after observing cystidia of *S. nikau* under a light microscope, that this ornamentation pattern was universal at the genus level. Regarding the shape of cystidia, Jülich (1975) noticed the less prominent basal swelling in *S. brachysporum* compared to *S. longisporum.* Oberwinkler (1977) characterised cystidia of *Subulicystidium* as uniform, but his remarks and especially illustrations displayed several deviations from the common pattern regarding both size and ornamentation. We further developed the idea of the importance of cystidial morphology and showed the presence of interspecific size differences as well as species-specific types of cystidial ornamentation (*S. oberwinkleri*, *S. harpagum*, *S. robustius*, *S. rarocrystallinum*). The important finding of Jülich (1975) is that the shape of single crystals can vary within a collection. The sharpness of the crystals is reduced with age, resulting in rounded instead of rectangular crystals as observed in a light microscope.

Beside cystidia, also hyphae and hymenial elements can have encrustation. Oberwinkler (1977) illustrated crystalline collars on the bases of basidia in the holotype of *S. meridense* and in the specimen of *S. brachysporum* sensu Boidin and Gilles (figs 30 and 29. respectively in Oberwinkler 1977) but not in other species. Kisimova-Horovitz et al. (1997) noticed a nearly ubiquitous presence of hymenial encrustation in *Subulicystidium*. We share this opinion after examining our collections. Jülich (1968) and Liberta (1980) observed repetobasidia and considered them to be a criterion of the genus *Subulicystidium*. On the contrary, Eriksson et al. (1984) did not observe any repetobasidia in the North European collections of *S. longisporum.* In our large set of tropical specimens we did not find any repetobasidia. Thus we suppose that repetobasidia observed by Jülich and Liberta are simply basidia with a well-developed crystalline collar.

Oberwinkler (1977) illustrated slightly thick-walled subicular hyphae in *S. meridense* but thin-walled in the rest of the species. We confirm this pattern for *S. meridense* but also observed similar deviating subicular hyphae in several other species, viz. *S. brachysporum*, *S. robustius*, *S. ryvardenii* and *S. oberwinkleri*. However, in all these cases, we found that the hyphal surface is rough and the wall is highly light-refractive, which means they are covered by crystalline material. In *S. harpagum*, this crystalline sheath around hyphae can reach a thickness of one and in *S. oberwinkleri* several micrometres. Under a light microscope, it is not possible to decide to what extent thickness depends on the cell wall or is due to the deposition of crystalline material.

Bourdot and Galzin (1912) reported a series of collections deviating from typical *S. longisporum* in fruit-body thickness and colouration and cystidial uniformity. The several varieties proposed by the French authors were not accepted by Jülich (1975) who considered them to represent normal variation and different developmental stages of *S. longisporum.* In line with Jülich (1975), after examining our specimens, we conclude that fruit-body thickness and density is variable within the species and thus of little value for the species-level identifications. Nevertheless, an experienced eye can differentiate the hirsute hymenial surface of the species with more robust cystidia (*S. robustius*, *S. ryvardenii*) from the velutinous hymenial surface of the rest of the genus. In a single species, *S. robustius*, we consistently observed slightly yellowish fruiting bodies, which was due to a yellowish hue of the hyphal and cystidial walls.

### Morphology complements molecular data for species delimitation

Partitioning sequence dissimilarity of both ITS and 28S into interspecific and intraspecific components revealed a clear barcode gap for some of the species but problems to delimit others. Therefore, both cases when morphology and available molecular information are congruent and cases when they are in conflict were found. This points to the importance of careful morphological examination and the need to combine morphology, rDNA barcode data and other DNA markers when defining species in *Subulicystidium*.

## Figures

**Figure 3.**
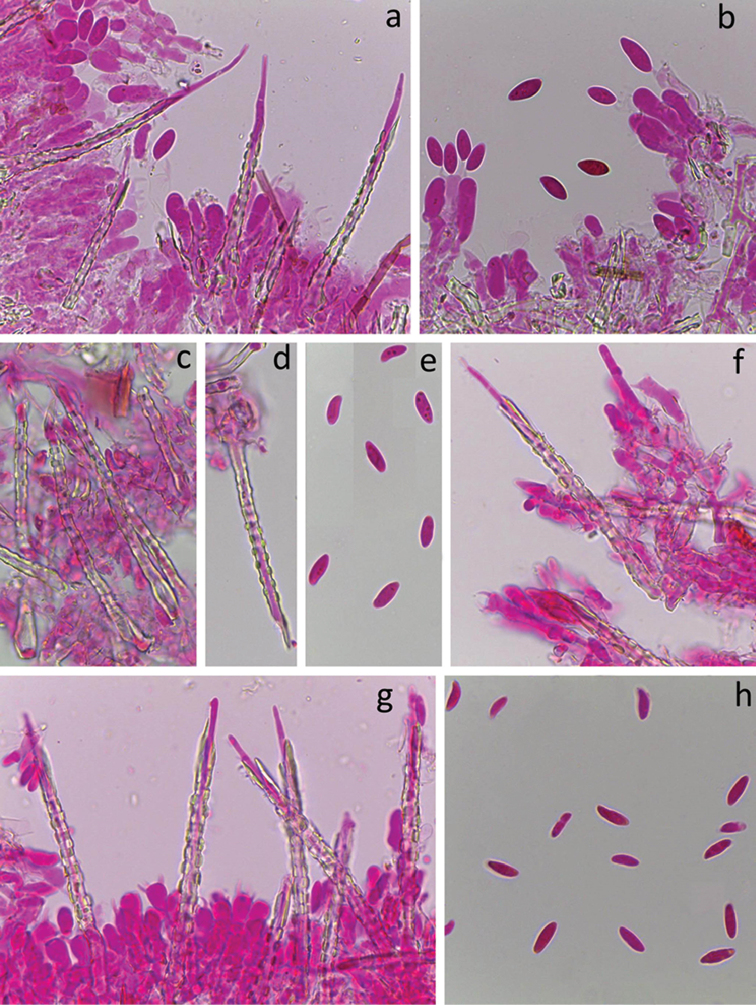
Species of *Subulicystidium* with broad fusiform basidiospores. *Subulicystidium naviculatum* (GB:KHL 11566): **a, b** hymenium and basidiospores. *Subulicystidium ryvardenii* (LR 8860/b in O:F 909583, holotype): **c, d** cystidia **e** basidiospores. *Subulicystidium robustius* (GB:KHL 10813, holotype): **f, g** cystidia in hymenium **h** basidiospores. All preparations done in 3% aqueous solution of potassium hydroxide (KOH) mixed with 1% aqueous solution of Phloxine. All scale bars equal 10 µm.

**Figure 5.**
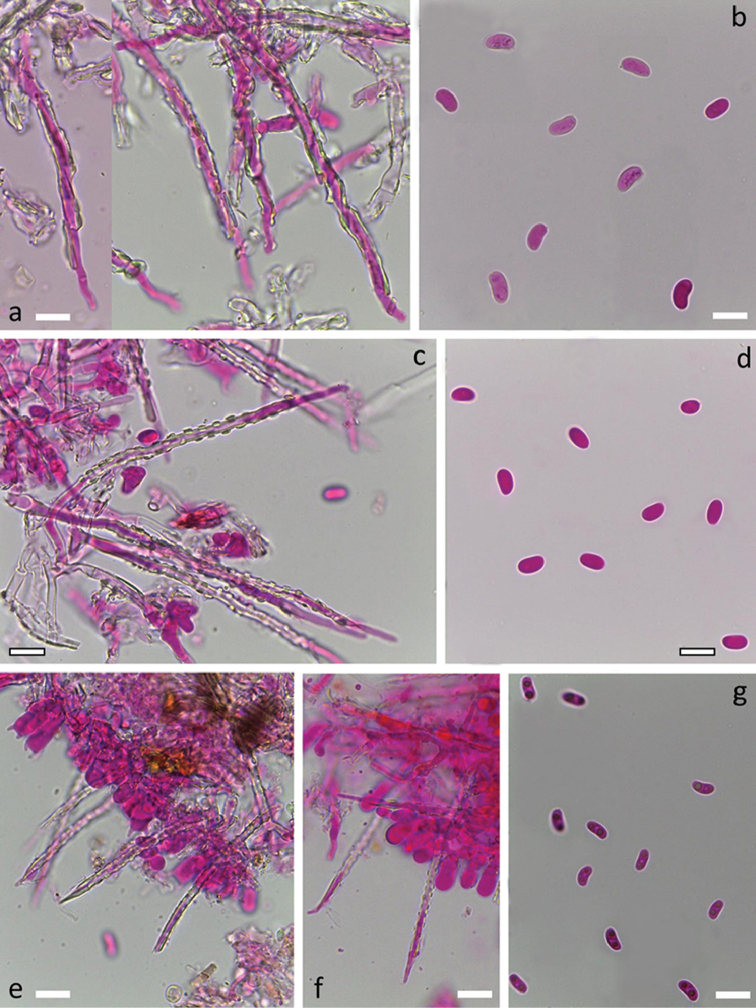
Species of *Subulicystidium* with broad cylindric basidiospores. *Subulicystidium oberwinkleri* (KAS:L 1860, holotype): **a** cystidia **b** basidiospores. *Subulicystidium nikau* (KAS:L 1296): **c** cystidia **d** basidiospores. *Subulicystidium boidinii* (KAS:L 1584a, holotype): **e** mature hymenium **f** young hymenium **g** basidiospores. All preparations done in 3% aqueous solution of potassium hydroxide (KOH) mixed with 1% aqueous solution of Phloxine. All scale bars equal 10 µm.

**Figure 6.**
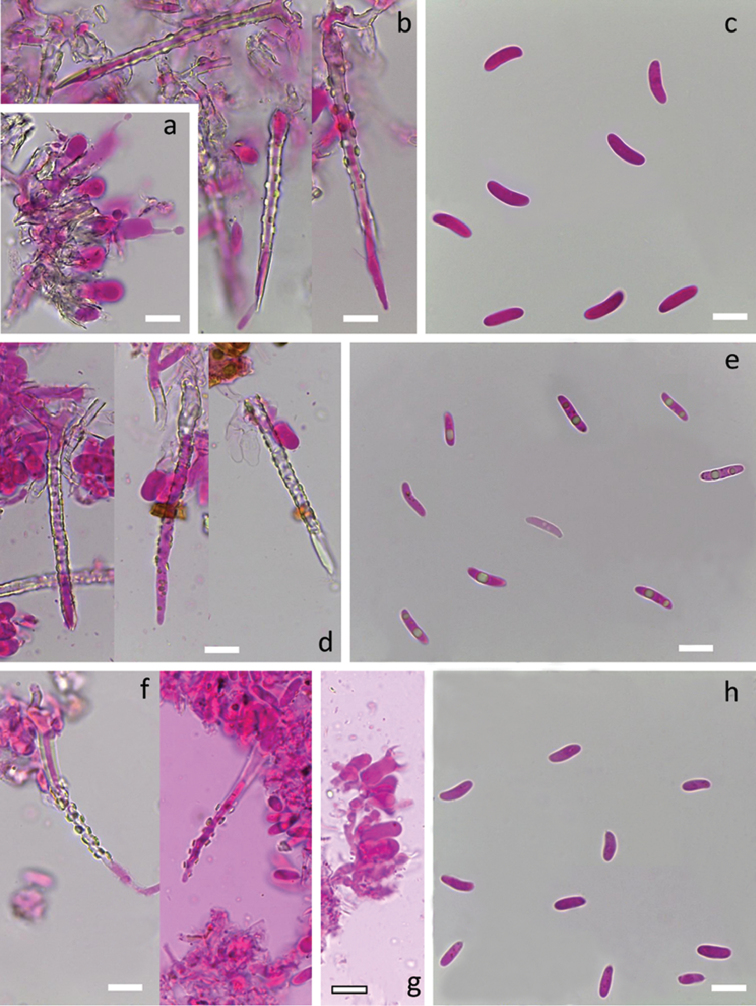
Species of *Subulicystidium* with long cylindric basidiospores. *Subulicystidium grandisporum* (LR 29162 in O:F 506781): **a** hymenium with rich crystalline encrustation **b** cystidia **c** basidiospores. *Subulicystidium obtusisporum* (FR: W213-3-I): **d** cystidia **e** basidiospores. *Subulicystidium rarocrystallinum* (LR 15483 in O:F 918488, holotype): **f** cystidia **g** hymenium **h** basidiospores. All preparations done in 3% aqueous solution of potassium hydroxide (KOH) mixed with 1% aqueous solution of Phloxine. All scale bars equal 10 µm.

**Figure 7.**
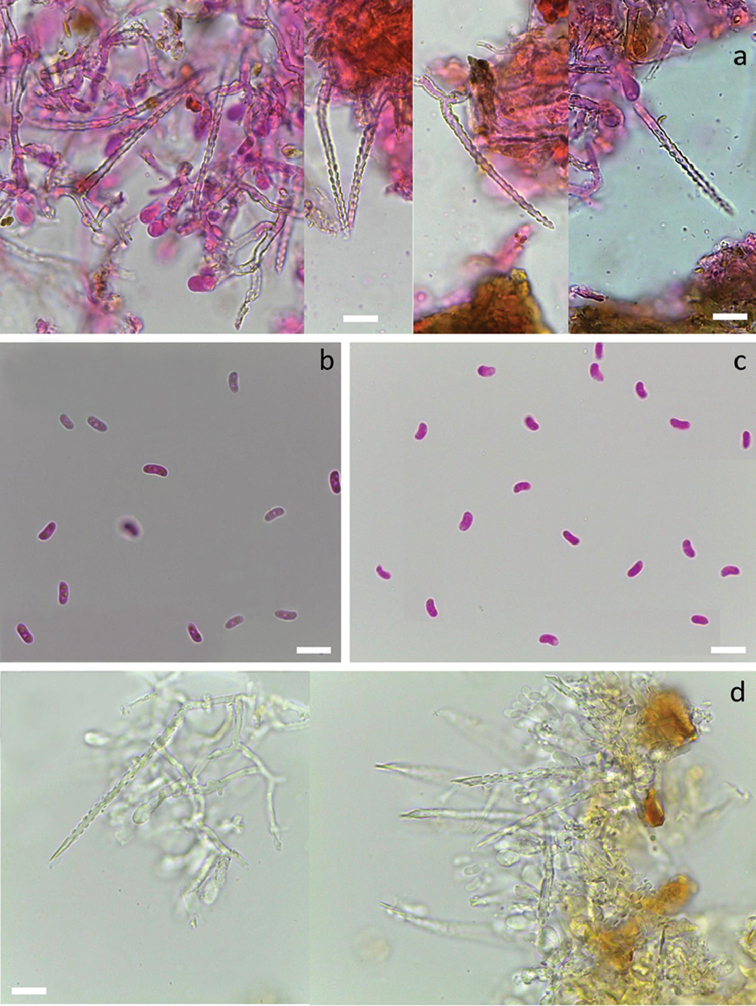
Species of *Subulicystidium* with smallest cylindric basidiospores. *Subulicystidium harpagum* (KAS:L 1726a, holotype): **a** cystidia **b** basidiospores. *Subulicystidium parvisporum* (KAS:L 0140, holotype): **c** basidiospores **d** cross sections through fruit-body. Preparations **a, b, c** done in 3% aqueous solution of potassium hydroxide (KOH) mixed with 1% aqueous solution of Phloxine, preparation **d** simply in KOH. All scale bars equal 10 µm.

**Figure 8.**
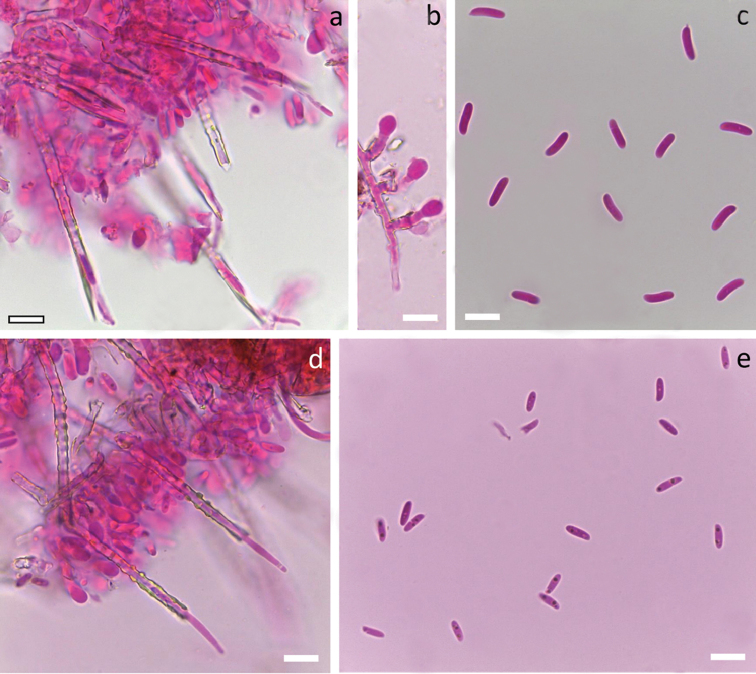
Species of *Subulicystidium brachysporum* morphotype. *Subulicystidium brachysporum* sensu Boidin and Gilles (LR 15784 in O:F 918493): **a** cystidia in hymenium **b** crystalline collars on basidioles and slightly encrustated subhymenial hyphae **c** basidiospores. *Subulicystidium brachysporum* sensu Talbot (LR 24170 in O:F): **d** cystidia in hymenium **e** basidiospores. All preparations done in 3% aqueous solution of potassium hydroxide (KOH) mixed with 1% aqueous solution of Phloxine. All scale bars equal 10 µm.

**Figure 9.**
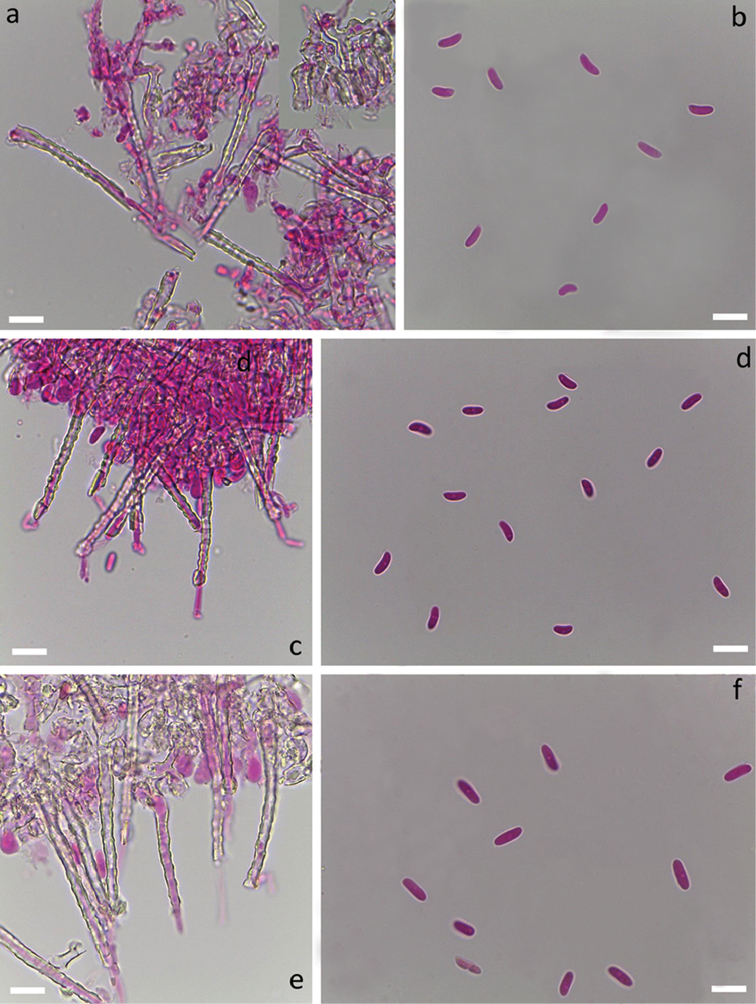
Species of *Subulicystidium meridense* morphotype. *Subulicystidium meridense*, holotype (TUB:FO 13761): **a** hymenium with crystalline encrustation and cystidia **b** basidiospores. *S. meridense* from own study (GB:KHL 11365): **c** cystidia in hymenium **d** basidiospores. Subulicystidium aff. meridense (GB:KHL 9561): **e** cystidia in hymenium **f** basidiospores. All preparations done in 3% aqueous solution of potassium hydroxide (KOH) mixed with 1% aqueous solution of Phloxine. All scale bars equal 10 µm.

## Supplementary Material

XML Treatment for Subulicystidium boidinii

XML Treatment for Subulicystidium fusisporum

XML Treatment for Subulicystidium grandisporum

XML Treatment for Subulicystidium harpagum

XML Treatment for Subulicystidium inornatum

XML Treatment for Subulicystidium oberwinkleri

XML Treatment for Subulicystidium parvisporum

XML Treatment for Subulicystidium rarocrystallinum

XML Treatment for Subulicystidium robustius

XML Treatment for Subulicystidium ryvardenii

XML Treatment for Subulicystidium tedersooi

## Supplementary materials

Supplementary material 1 Detailed data on 144 specimens of *Subulicystidium* used in the study Data type: occurences Explanation note: Specimens for which coordinates were available on herbarium labels or from field notes contain “gps” in the column “COORD_source”. Specimens for which coordinates were estimated from the map web sources (see Materials and Methods) are marked with the word “map”. This is an open access article distributed under the terms of the Creative Commons Attribution License (CC BY 4.0), which permits unrestricted use, distribution, and reproduction in any medium, provided the original author and source are credited.Alexander Ordynets, David Scherf, Felix Pansegrau, Jonathan Denecke, Ludmila Lysenko, Karl-Henrik Larsson, Ewald Langer

Supplementary material 2 Measurements of 2840 basidiospores from 67 *Subulicystidium* specimens which were sequenced or represent important historical collections Data type: morphological Explanation note: Each row contains data on length (L_sp), width (W_sp) and length to width ratio (Q_sp) of a single basidiospore and information on specimen and species from which the basidiospore was measured is provided in separate columns. Dataset does not contain outliers which were found for some specimens in raw data (see Materials and methods for details of excluding outliers). In the case of *S. brachysporum*, the capital “B” following epithet means morphological species concept following Boidin and Gilles (1988), while “T” means the species as described by Talbot (1958). This is an open access article distributed under the terms of the Creative Commons Attribution License (CC BY 4.0), which permits unrestricted use, distribution, and reproduction in any medium, provided the original author and source are credited.Alexander Ordynets, David Scherf, Felix Pansegrau, Jonathan Denecke, Ludmila Lysenko, Karl-Henrik Larsson, Ewald Langer

Supplementary material 3 Basidiospore size ranges of 67 *Subulicystidium* specimens which were sequenced or represent important historical collections Data type: morphological Explanation note: For the parameters of basidiospore length (L), width (W) and length to width ratio (Q), the following values are presented: minimal value, 5% data quantile, mean, 95% data quantile and maximum. Minimum and maximum do not consider outliers which were found for some specimens in raw data (see Materials and methods for details of excluding outliers). This is an open access article distributed under the terms of the Creative Commons Attribution License (CC BY 4.0), which permits unrestricted use, distribution, and reproduction in any medium, provided the original author and source are credited.Alexander Ordynets, David Scherf, Felix Pansegrau, Jonathan Denecke, Ludmila Lysenko, Karl-Henrik Larsson, Ewald Langer

Supplementary material 4 Basidiospore size ranges of *Subulicystidium* species included in the study Data type: morphological Explanation note: Calculations are based on a set of 67 specimens which were sequenced or represent important historical collections. For the parameters of basidiospore length (L), width (W) and length to width ratio (Q), the following values are presented: minimal value, 5% data quantile, mean, 95% data quantile and maximum. Minimum and maximum do not consider outliers which were found for some specimens in raw data (see Materials and methods for details of excluding outliers). Calculations were provided separately for the type specimens (of *S. meridense* and *S. nikau*), as well as for the collection as *S. boidinii* LY 11247 which was used to propose “*Subulicystidium allantosporum* ad interim” (Boidin and Gilles 1988). In *S. brachysporum*, the capital “B” following epithet means morphological species concept following Boidin and Gilles (1988), while “T” means the species as described by Talbot (1958). This is an open access article distributed under the terms of the Creative Commons Attribution License (CC BY 4.0), which permits unrestricted use, distribution, and reproduction in any medium, provided the original author and source are credited.Alexander Ordynets, David Scherf, Felix Pansegrau, Jonathan Denecke, Ludmila Lysenko, Karl-Henrik Larsson, Ewald Langer

## References

[B1] Abarenkov K Tedersoo L Nilsson RH Vellak K Saar I Veldre V Parmasto E Prous M Aan A Ots M Kurina O Ostonen I Jõgeva J Halapuu S Põldmaa K Toots M Truu J Larsson K-H Kõljalg U (2010) PlutoF–a Web Based Workbench for Ecological and Taxonomic Research, with an Online Implementation for Fungal ITS Sequences. Evolutionary Bioinformatics Online 6: 189–196. https://doi.org/10.4137/EBO.S6271

[B2] Bengtsson-Palme J Ryberg M Hartmann M Branco S Wang Z Godhe A De Wit P Sánchez-García M Ebersberger I de Sousa F Amend AS Jumpponen A Unterseher M Kristiansson E Abarenkov K Bertrand YJK Sanli K Eriksson KM Vik U Veldre V Nilsson RH (2013) Improved software detection and extraction of ITS1 and ITS2 from ribosomal ITS sequences of fungi and other eukaryotes for analysis of environmental sequencing data Bunce M (Ed). Methods in Ecology and Evolution 4: 914–919. https://doi.org/10.1111/2041-210X.12073

[B3] Boidin J Gilles G (1988) Basidiomycètes aphyllophorales de l’Ile de la Réunion. XII: Le genre Subulicystidium Parmasto. Bulletin trimestriel de la Société mycologique de France 104: 191–198. Available from: http://cat.inist.fr/?aModele=afficheN&cpsidt=7053739 [November 10, 2014]

[B4] Bourdot H Galzin A (1912) Hyménomycètes de France: IV. Corticiées: Vuilleminia, Aleurodiscus, Dendrothele, Gloeocystidium, Peniophora. Bulletin de la Société Mycologique de France 28: 349–409.

[B5] Brown SDJ Collins RA Boyer S Lefort MC Malumbres-Olarte J Vink CJ Cruickshank RH (2012) Spider: An R package for the analysis of species identity and evolution, with particular reference to DNA barcoding. Molecular Ecology Resources 12: 562–565. https://doi.org/10.1111/j.1755-0998.2011.03108.x 22243808 10.1111/j.1755-0998.2011.03108.x

[B6] Calhim S Halme P Petersen JH Læssøe T Bässler C Heilmann-Clausen J (2018) Fungal spore diversity reflects substrate-specific deposition challenges. Scientific Reports 8: 5356. https://doi.org/10.1038/s41598-018-23292-8 10.1038/s41598-018-23292-8 PMC5876365 29599480

[B7] Collins RA Cruickshank RH (2012) The seven deadly sins of DNA barcoding. Molecular Ecology Resources 13: 969–975. https://doi.org/10.1111/1755-0998.12046 23280099 10.1111/1755-0998.12046

[B8] Collopy PD Largeteau-Mamoun ML Romaine CP Royse DJ (2001) Molecular Phylogenetic Analyses of *Verticillium fungicola* and Related Species Causing Dry Bubble Disease of the Cultivated Button Mushroom, *Agaricus bisporus*. Phytopathology 91: 905–912. https://doi.org/10.1094/PHYTO.2001.91.9.905 18944237 10.1094/PHYTO.2001.91.9.905

[B9] Cunningham GH (1955) Thelephoraceae of New Zealand. Part VI. The genus Peniophora. Transactions and Proceedings of the Royal Society of New Zealand 83: 247–293.

[B10] David HA Hartley HO Pearson ES (1954) The Distribution of the Ratio, in a Single Normal Sample, of Range to Standard Deviation. Biometrika 41: 482. https://doi.org/10.2307/2332728

[B11] Duhem B Michel H (2001) Contribution à la connaissance du genre Subulicystidium Parmasto 1968 (Basidiomycota, Xenasmatales). Cryptogamie Mycologie 22: 163–173. https://doi.org/10.1016/S0181-1584(01)01067-3

[B12] Edgar RC (2004) MUSCLE: multiple sequence alignment with high accuracy and high throughput. Nucleic Acids Research 32: 1792–1797. https://doi.org/10.1093/nar/gkh340 15034147 10.1093/nar/gkh340 PMC390337

[B13] Eriksson J Hjortstam K Ryvarden L (1984) The Corticiaceae of North Europe 7. Fungiflora, Oslo, 1282–1449.

[B14] Galante TE Horton TR Swaney DP (2011) 95% of basidiospores fall within 1 m of the cap: a field-and modeling-based study. Mycologia 103: 1175–1183. https://doi.org/10.3852/10-388 21700637 10.3852/10-388

[B15] Gardes M Bruns TD (1993) ITS primers with enhanced specificity for basidiomycetes, application to the identification of mycorrihiza and rusts. Molecular Ecology 2: 113–118. https://doi.org/10.1111/J.1365-294x.1993.Tb00005.X 8180733 10.1111/j.1365-294x.1993.tb00005.x

[B16] Geml J Timling I Robinson CH Lennon N Nusbaum HC Brochmann C Noordeloos ME Taylor DL (2012) An arctic community of symbiotic fungi assembled by long-distance dispersers: phylogenetic diversity of ectomycorrhizal basidiomycetes in Svalbard based on soil and sporocarp DNA. Journal of Biogeography 39: 74–88. https://doi.org/10.1111/j.1365-2699.2011.02588.x

[B17] Ghobad-Nejhad M Hallenberg N Parmasto E Kotiranta H (2009) A first annotated checklist of corticioid and polypore basidiomycetes of the Caucasus region. Mycologia Balcanica 6: 123–168.

[B18] Gorjón SP Greslebin AG Rajchenberg M (2012) Subulicystidium curvisporum sp. nov. (Hymenochaetales, Basidiomycota) from the Patagonian Andes. Mycotaxon 118: 47–52. https://doi.org/10.5248/118.47

[B19] Gouy M Guindon S Gascuel O (2010) SeaView version 4: A multiplatform graphical user interface for sequence alignment and phylogenetic tree building. Molecular biology and evolution 27: 221–224. https://doi.org/10.1093/molbev/msp259 19854763 10.1093/molbev/msp259

[B20] Grubbs FE (1950) Sample Criteria for Testing Outlying Observations. The Annals of Mathematical Statistics 21: 27–58. https://doi.org/10.1214/aoms/1177729885

[B21] Hibbett DS Bauer R Binder M Giachini AJ Hosaka K Justo A Larsson E Larsson KH Lawrey JD Miettinen O Nagy LG Nilsson RH Weiss M Thorn RG (2014) 14 Agaricomycetes. In: McLaughlin DJ Spatafora JW (Eds) Systematics and Evolution. The Mycota VII Part A. Springer, Berlin, Heidelberg, 373–429. https://doi.org/10.1007/978-3-642-55318-9_14

[B22] Hjortstam K Ryvarden L (1986) Some new and noteworthy fungi (Aphyllophorales, Basidiomycetes) from Iguazu, Argentina. Mycotaxon 25: 539–567.

[B23] Hopple JS Vilgalys R (1999) Phylogenetic Relationships in the Mushroom Genus Coprinus and Dark-Spored Allies Based on Sequence Data from the Nuclear Gene Coding for the Large Ribosomal Subunit RNA: Divergent Domains, Outgroups, and Monophyly. Molecular Phylogenetics and Evolution 13: 1–19. http://dx.doi.org/10.1006/mpev.1999.0634 10508535 10.1006/mpev.1999.0634

[B24] Howe WG (1969) Two-Sided Tolerance Limits for Normal Populations, Some Improvements. Journal of the American Statistical Association 64: 610. https://doi.org/10.2307/2283644

[B25] Index Fungorum (2018) Index Fungorum. http://www.indexfungorum.org [April 26, 2018]

[B26] Izumitsu K Hatoh K Sumita T Kitade Y Morita A Gafur A Ohta A Kawai M Yamanaka T Neda H Ota Y Tanaka C (2012) Rapid and simple preparation of mushroom DNA directly from colonies and fruiting bodies for PCR. Mycoscience 53: 396–401. https://doi.org/10.1007/s10267-012-0182-3

[B27] Jülich W (1968) Über die Gattungen Piloderma gen. nov. und Subulicystidium Parm. Berichte der Deutschen Botanischen Gesellschaft 81: 414–421. https://doi.org/10.1111/J.1438-8677.1969.TB02159.X 11637081

[B28] Jülich W (1975) Studien an Cystiden-I. Subulicystidium Parm. Persoonia 8: 187–190.

[B29] Katoh K Rozewicki J Yamada KD (2017) MAFFT online service: multiple sequence alignment, interactive sequence choice and visualization. Briefings in Bioinformatics. https://doi.org/10.1093/bib/bbx108 10.1093/bib/bbx108 PMC6781576 28968734

[B30] Kauserud H Schumacher T (2001) Outcrossing or inbreeding: DNA markers provide evidence for type of reproductive mode in *Phellinus nigrolimitatus* (Basidiomycota). Mycological Research 105: 676–683. https://doi.org/10.1017/S0953756201004191

[B31] Kearse M Moir R Wilson A Stones-Havas S Cheung M Sturrock S Buxton S Cooper A Markowitz S Duran C Thierer T Ashton B Meintjes P Drummond A (2012) Geneious Basic: An integrated and extendable desktop software platform for the organization and analysis of sequence data. Bioinformatics 28: 1647–1649. https://doi.org/10.1093/bioinformatics/bts199 22543367 10.1093/bioinformatics/bts199 PMC3371832

[B32] Keller J (1985) Les cystides cristalliferes des Aphyllophorales. Mycologia Helvetica 1: 277–340.

[B33] Kisimova-Horovitz L Oberwinkler F Gómez-Pignataro LD (1997) Basidiomicetos resupinados de Costa Rica. *Litschauerella*, Subulicystidium y *Tubulicium* (Corticiaceae sl). Resupinate basidiomycetes from Costa Rica. *Litschauerella*, Subulicystidium and *Tubulicium* (Corticiaceae sl). Revista de Biología Tropical. 45: 1311–1325.

[B34] Kõljalg U Nilsson RH Abarenkov K Tedersoo L Taylor AFS Bahram M Bates ST Bruns TD Bengtsson-Palme J Callaghan TM Douglas B Drenkhan T Eberhardt U Dueñas M Grebenc T Griffith GW Hartmann M Kirk PM Kohout P Larsson E Lindahl BD Lücking R Martín MP Matheny PB Nguyen NH Niskanen T Oja J Peay KG Peintner U Peterson M Põldmaa K Saag L Saar I Schüßler A Scott JA Senés C Smith ME Suija A Taylor DL Telleria MT Weiss M Larsson K (2013) Towards a unified paradigm for sequence-based identification of fungi. Molecular Ecology 22: 5271–5277. https://doi.org/10.1111/mec.12481 24112409 10.1111/mec.12481

[B35] Larsson A (2014) AliView: a fast and lightweight alignment viewer and editor for large datasets. Bioinformatics 30: 3276–3278. https://doi.org/10.1093/bioinformatics/btu531 25095880 10.1093/bioinformatics/btu531 PMC4221126

[B36] Larsson K-H (2007) Re-thinking the classification of corticioid fungi. Mycological Research 111: 1040–1063. https://doi.org/10.1016/j.mycres.2007.08.001 17981020 10.1016/j.mycres.2007.08.001

[B37] Liberta AE (1980) Notes on the genus Subulicystidium. Mycotaxon 10: 409–412.

[B38] Maddison WP Maddison DR (2018) Mesquite: a modular system for evolutionary analysis. Version 3.40. http://mesquiteproject.org

[B39] Maekawa N (2002) Corticioid fungi (Basidiomycota) collected in Vanuatu. Annals of the Tsukuba Botanical Garden 21: 119–126. https://ci.nii.ac.jp/els/contents110004697627.pdf?id=ART0007436943

[B40] Martini E (2016) Excerpts from Crusts & Jells. Descriptions and reports of resupinate Aphyllophorales and Heterobasidiomycetes Issue № 13: Subulicystidium brachysporum Bignasco. https://www.aphyllo.net/spec.php?id=1410300

[B41] Matheny PB Liu YJ Ammirati JF Hall BD (2002) Using RPB1 sequences to improve phylogenetic inference among mushrooms (Inocybe, Agaricales). American Journal of Botany 89: 688–698. https://doi.org/10.3732/ajb.89.4.688 21665669 10.3732/ajb.89.4.688

[B42] Meyer CP Paulay G Hartl D Hewitt G Petersen G (2005) DNA Barcoding: Error Rates Based on Comprehensive Sampling Godfray C (Ed). PLoS Biology 3: e422. https://doi.org/10.1371/journal.pbio.0030422 10.1371/journal.pbio.0030422 PMC1287506 16336051

[B43] Miller MA Pfeiffer W Schwartz T (2010) Creating the CIPRES Science Gateway for inference of large phylogenetic trees. In: 2010 Gateway Computing Environments Workshop (GCE). IEEE, 1–8. https://doi.org/10.1109/GCE.2010.5676129

[B44] Nilsson RH Tedersoo L Abarenkov K Ryberg M Kristiansson E Hartmann M Schoch CL Nylander JAA Bergsten J Porter TM Jumpponen A Vaishampayan P Ovaskainen O Hallenberg N Bengtsson-Palme J Eriksson KM Larsson K-H Larsson E Kõljalg U (2012) Five simple guidelines for establishing basic authenticity and reliability of newly generated fungal ITS sequences. MycoKeys 4: 37–63. https://doi.org/10.3897/mycokeys.4.3606

[B45] Nordén B Larsson K-H (2000) Basidiospore dispersal in the old-growth forest fungus Phlebia centrifuga (Basidiomycetes). Nordic Journal of Botany 20: 215–219. https://doi.org/10.1111/j.1756-1051.2000.tb01572.x

[B46] Norros V Penttilä R Suominen M Ovaskainen O (2012) Dispersal may limit the occurrence of specialist wood decay fungi already at small spatial scales. Oikos 121: 961–974. https://doi.org/10.1111/j.1600-0706.2012.20052.x

[B47] Norros V Rannik Ü Hussein T Petäjä T Vesala T Ovaskainen O (2014) Do small spores disperse further than large spores? Ecology 95: 1612–1621. https://doi.org/10.1890/13-0877.1 10.1890/13-0877.1 25039225

[B48] O’Donnell K (1992) Fusarium and its near relatives. The fungal holomorph: mitotic, meiotic and pleomorphic speciation in fungal systematics. CAB International, Wallingford, pp 225-233.

[B49] Oberwinkler F (1977) Species and generic concepts in the Corticiaceae The species concept in Hymenomycetes. Cramer, Vaduz: 331–348.

[B50] Ordynets A (2018a) Calculating and plotting genetic distances. https://www.protocols.io http://doi.org/10.17504/protocols.io.n8fdhtn

[B51] Ordynets A (2018b) Calculating tolerance intervals for the size of measurable objects. https://www.protocols.io http://doi.org/10.17504/protocols.io.n8gdhtw

[B52] Ordynets A (2018c) Estimating barcoding gap for species delimitation. https://www.protocols.io http://doi.org/10.17504/protocols.io.n98dh9w

[B53] Ordynets A (2018d) Plotting phylogenetic tree with branch supports from two phylogenetic analyses. https://www.protocols.io http://doi.org/10.17504/protocols.io.n9fdh3n

[B54] Ordynets A Denecke J (2018) Calculating and plotting size range of morphological structures. https://www.protocols.io http://doi.org/10.17504/protocols.io.n7tdhnn

[B55] Ordynets A Larsson K-H Langer E (2015) Two new *Trechispora* species from La Réunion Island. Mycological Progress 14: 113. https://doi.org/10.1007/s11557-015-1133-0

[B56] Paradis E Claude J Strimmer K (2004) APE: Analyses of phylogenetics and evolution in R language. Bioinformatics 20: 289–290. https://doi.org/10.1093/bioinformatics/btg412 14734327 10.1093/bioinformatics/btg412

[B57] Parmasto E Parmasto I Möls T (1987) Variation of basidiospores in the hymenomycetes and its significance to their taxonomy. Bresinsky A, Butin H, Schwantes HO (Eds) J. Cramer, Berlin, Stuttgart.

[B58] Peay KG Bidartondo MI Elizabeth Arnold A (2010) Not every fungus is everywhere: Scaling to the biogeography of fungal-plant interactions across roots, shoots and ecosystems. New Phytologist 185: 878–882. https://doi.org/10.1111/j.1469-8137.2009.03158.x 20356342 10.1111/j.1469-8137.2009.03158.x

[B59] Peay KG Schubert MG Nguyen NH Bruns TD (2012) Measuring ectomycorrhizal fungal dispersal: macroecological patterns driven by microscopic propagules. Molecular Ecology 21: 4122–4136. https://doi.org/10.1111/j.1365-294X.2012.05666.x 22703050 10.1111/j.1365-294X.2012.05666.x

[B60] Punugu A Dunn MT Welden AL (1980) Peniophoroid fungi of the West Indies. Mycotaxon 10: 428–454.

[B61] R Core Team (2017) R: A Language and Environment for Statistical Computing. Available from: https://www.r-project.org/

[B62] Raitviir A (1972) Statistical methods and species delimitation in the genus Otidea. Persoonia 6: 415–423. http://www.repository.naturalis.nl/record/532069 (April 27, 2017).

[B63] Rambaut A (2014) FigTree, a graphical viewer of phylogenetic trees. http://tree.bio.ed.ac.uk/software/figtree/

[B64] Rambaut A Suchard MA Xie D Drummond AJ (2014) Tracer v1.6. http://beast.bio.ed.ac.uk/Tracer.

[B65] Ronquist F Huelsenbeck J Teslenko M (2011) Draft MrBayes version 3.2 manual: tutorials and model summaries. Distributed with the software from http://brahms. biology.rochester. edu/software. html

[B66] Ronquist F Teslenko M van der Mark P Ayres DL Darling A Höhna S Larget B Liu L Suchard MA Huelsenbeck JP (2012) MrBayes 3.2: efficient Bayesian phylogenetic inference and model choice across a large model space. Systematic biology 61: 539–542. Available from: http://sysbio.oxfordjournals.org/content/61/3/539.short. 10.1093/sysbio/sys029 PMC3329765 22357727

[B67] Rüdigs J (Makroaufmaßprogramm. https://ruedig.de/tmp/messprogramm.htm [April 10, 2018]

[B68] Schoch CL Seifert KA Huhndorf S Robert V Spouge JL Levesque CA Chen W Bolchacova E Voigt K Crous PW Miller AN Wingfield MJ Aime MC An K-D Bai F-Y Barreto RW Begerow D Bergeron M-J Blackwell M Boekhout T Bogale M Boonyuen N Burgaz AR Buyck B Cai L Cai Q Cardinali G Chaverri P Coppins BJ Crespo A Cubas P Cummings C Damm U de Beer ZW de Hoog GS Del-Prado R Dentinger B Dieguez-Uribeondo J Divakar PK Douglas B Duenas M Duong TA Eberhardt U Edwards JE Elshahed MS Fliegerova K Furtado M Garcia MA Ge Z-W Griffith GW Griffiths K Groenewald JZ Groenewald M Grube M Gryzenhout M Guo L-D Hagen F Hambleton S Hamelin RC Hansen K Harrold P Heller G Herrera C Hirayama K Hirooka Y Ho H-M Hoffmann K Hofstetter V Hognabba F Hollingsworth PM Hong S-B Hosaka K Houbraken J Hughes K Huhtinen S Hyde KD James T Johnson EM Johnson JE Johnston PR Jones EBG Kelly LJ Kirk PM Knapp DG Koljalg U Kovacs GM Kurtzman CP Landvik S Leavitt SD Liggenstoffer AS Liimatainen K Lombard L Luangsa-ard JJ Lumbsch HT Maganti H Maharachchikumbura SSN Martin MP May TW McTaggart AR Methven AS Meyer W Moncalvo J-M Mongkolsamrit S Nagy LG Nilsson RH Niskanen T Nyilasi I Okada G Okane I Olariaga I Otte J Papp T Park D Petkovits T Pino-Bodas R Quaedvlieg W Raja HA Redecker D Rintoul TL Ruibal C Sarmiento-Ramirez JM Schmitt I Schussler A Shearer C Sotome K Stefani FOP Stenroos S Stielow B Stockinger H Suetrong S Suh S-O Sung G-H Suzuki M Tanaka K Tedersoo L Telleria MT Tretter E Untereiner WA Urbina H Vagvolgyi C Vialle A Vu TD Walther G Wang Q-M Wang Y Weir BS Weiss M White MM Xu J Yahr R Yang ZL Yurkov A Zamora J-C Zhang N Zhuang W-Y Schindel D (2012) Nuclear ribosomal internal transcribed spacer (ITS) region as a universal DNA barcode marker for Fungi. Proceedings of the National Academy of Sciences 109: 6241–6246. https://doi.org/10.1073/pnas.1117018109 10.1073/pnas.1117018109 PMC3341068 22454494

[B69] Senckenberg. Herbarium Senckenbergianum (FR) – Fungi (2018) Occurrence Dataset https://doi.org/10.15468/0oaq5v [accessed via GBIF.org on 2018-03-28]

[B70] Stalpers JA Buchanan PK (1991) Type studies of the species of *Pellicularia* and Peniophora described by G.H. Cunningham. New Zealand Journal of Botany 29: 331–340.

[B71] Stamatakis A (2014) RAxML version 8: A tool for phylogenetic analysis and post-analysis of large phylogenies. Bioinformatics 30: 1312–1313. https://doi.org/10.1093/bioinformatics/btu033 24451623 10.1093/bioinformatics/btu033 PMC3998144

[B72] Stamatakis A (2016) The RAxML v8.2.X Manual. http://sco.h-its.org/exelixis/resource/download/NewManual.pdf [December 14, 2016]

[B73] Subulicystidium longisporum (Pat.) Parmasto in GBIF Secretariat (2017) GBIF Backbone Taxonomy. Checklist Dataset https://doi.org/10.15468/39omei [accessed via GBIF.org on 2018-03-28] https://www.gbif.org/species/2523214

[B74] Talbot PHB (1958) Studies of some South African resupinate Hymenomycetes. Part II. Bothalia 7: 131–187.

[B75] Telleria MT Melo I Duenas M Larsson K-H Martín MPP (2013) Molecular analyses confirm Brevicellicium in Trechisporales. IMA Fungus 4: 21–28. https://doi.org/10.5598/imafungus.2013.04.01.03 23898409 10.5598/imafungus.2013.04.01.03 PMC3719203

[B76] Verma SP Quiroz-Ruiz A (2006) Critical values for six Dixon tests for outliers Critical values for six Dixon tests for outliers in normal samples up to sizes 100, and applications in science and engineering. Revista Mexicana de Ciencias Geológicas 23: 133–161. http://scielo.unam.mx/pdf/rmcg/v23n2/v23n2a3.pdf [January 11, 2017]

[B77] Volobuev S (2016) Subulicystidium perlongisporum (Trechisporales, Basidiomycota) new to Russia, with notes on a molecular study of the species. Nova Hedwigia 102: 531–537. https://doi.org/10.1127/nova_hedwigia/2016/0329

[B78] White TJ Bruns S Lee S Taylor J (1990) Amplification and direct sequencing of fungal ribosomal RNA genes for phylogenetics. In: PCR Protocols: A Guide to Methods and Applications. Academic Press, New York, 315–322. doi: citeulike-article-id:671166

[B79] Wickham H (2009) ggplot2: elegant graphics for data analysis. Springer, New York. https://doi.org/10.1007/978-0-387-98141-3

[B80] Wieczorek C Wieczorek J (2015) Georeferencing Calculator (version 20160929). Museum of Vertebrate Zoology, University of California, Berkeley. Available: http://manisnet.org/gci2.html [Accessed 2018-03-28]

[B81] Wilk J (2012) Smaff – “Statistische Messreihen-Auswertung für Fungi v3.1. ” Südwestdeutsche Pilzrundschau 48: 49–56. http://www.pilzfreun.de/zeitschrift/

[B82] Young DS (2010) tolerance: An R Package for Estimating Tolerance Intervals. Journal of Statistical Software 36: 1–39. https://doi.org/10.18637/jss.v036.i05

[B83] Yurchenko E Wu S-H (2016) A key to the species of *Hyphodontia* sensu lato. MycoKeys 12: 1–27. https://doi.org/10.3897/mycokeys.12.7568

